# Effective conductivity and permittivity of unsaturated porous materials in the frequency range 1 mHz–1GHz

**DOI:** 10.1029/2012WR012700

**Published:** 2013-01-24

**Authors:** A Revil

**Affiliations:** 1 Department of Geophysics, Colorado School of Mines, Green CenterGolden, Colorado, USA; 2 ISTerre, CNRS, UMR CNRS 5275, Université de SavoieLe Bourget du Lac, France

## Abstract

A model combining low-frequency complex conductivity and high-frequency permittivity is developed in the frequency range from 1 mHz to 1 GHz. The low-frequency conductivity depends on pore water and surface conductivities. Surface conductivity is controlled by the electrical diffuse layer, the outer component of the electrical double layer coating the surface of the minerals. The frequency dependence of the effective quadrature conductivity shows three domains. Below a critical frequency *f_p_*, which depends on the dynamic pore throat size Λ, the quadrature conductivity is frequency dependent. Between *f_p_* and a second critical frequency *f_d_*, the quadrature conductivity is generally well described by a plateau when clay minerals are present in the material. Clay-free porous materials with a narrow grain size distribution are described by a Cole-Cole model. The characteristic frequency *f_d_* controls the transition between double layer polarization and the effect of the high-frequency permittivity of the material. The Maxwell-Wagner polarization is found to be relatively negligible. For a broad range of frequencies below 1 MHz, the effective permittivity exhibits a strong dependence with the cation exchange capacity and the specific surface area. At high frequency, above the critical frequency *f_d_*, the effective permittivity reaches a high-frequency asymptotic limit that is controlled by the two Archie's exponents *m* and *n* like the low-frequency electrical conductivity. The unified model is compared with various data sets from the literature and is able to explain fairly well a broad number of observations with a very small number of textural and electrochemical parameters. It could be therefore used to interpret induced polarization, induction-based electromagnetic methods, and ground penetrating radar data to characterize the vadose zone.

## 1. Introduction

There are a number of electrical and electromagnetic (EM) geophysical methods providing nonintrusively information relative to the texture, water content, and interfacial electrochemistry in the shallow subsurface in an extremely broad range of frequencies (1 mHz–1 GHz). The relevant arsenal of nonintrusive techniques include self-potential, direct current (DC) resistivity, frequency and time-domain induced polarization, frequency and time-domain induction EM methods, ground penetrating radar (GPR), and the seismoelectric/electroseismic methods. These nonintrusive methods have many applications in hydrogeophysics to assess ground water resources and to monitor ground water quality or for vadose zone hydrogeology and agriculture just to cite few examples [e.g., *Linde et al*., 2006]. That said, there is not a single model able to explain the complex conductivity of unsaturated materials as well as their effective permittivity and their frequency dependences in the broad range of frequencies of interest in geophysics (1 mHz–1 GHz).

In the low-frequency domain (<10 kHz), new models have been developed recently to describe the complex conductivity of saturated and unsaturated sands and clayey materials [*Revil and Florsch*, 2010; *Revil*, 2012; *Revil et al*., 2012a, 2012b]. There is a vast literature regarding this topic in interfacial electrochemistry and colloidal chemistry [e.g., *Schwarz* 1962; *Dukhin and Shilov*, 2002; *Grosse*, 2002] as well as in geophysics [*Tarasov and Titov*, 2007]. At very high frequencies, there are also predictive models describing the high-frequency permittivity of porous rocks. These models are empirically based like the Topp model for soils [*Topp and Reynolds*, 1998], based on upscaling methods like the volume-averaging method [*Pride*, 1994], or based on the effective differential medium approach or mixing laws (see discussion in *Jones and Or* [2002], *Cosenza et al*. [2003], *Robinson and Friedman* [2003], and *Miyamoto et al*. [2005]). Note that with the volume-averaging approach, the different phases are considered symmetrically while with the differential effective medium approach, one phase has to be chosen as the host (for instance, grains immersed in a background fluid or fluid inclusion in a matrix).

The situation is much more confused in the intermediate range of frequencies (10 kHz–10 MHz). Typically, a number of authors in geophysics have extended the high-frequency behavior to lower frequencies by adding a contribution related to the Maxwell-Wagner polarization mechanism [*Chen and Or*, 2006]. Other approaches have relied on using the so-called standard model of colloidal chemistry (developed initially by Dukhin and Shilov and based on the polarization of the diffuse layer, see *Grosse* [2002]) and applying this model to low-frequency data sets [*Garrouch and Sharma*, 1994]. To fit the data, a stretch of the textural parameters is usually required and the obtained set of interfacial properties are not compatible with the triple layer complexation models commonly used in surface electrochemistry of minerals.

In this paper, following my previous works [*Revil and Florsch*, 2010; *Revil*, 2012; *Revil et al*., 2012a, 2012b], I claim that the diffuse layer is not the main contributor to low-frequency polarization of clayey materials. I also claim that the Maxwell-Wagner polarization is not either the dominating mechanism of polarization at intermediate frequencies. An extremely simple model is developed based on the low-frequency polarization of the Stern layer (the inner layer of the electrical double layer). This model is proposed for saturated and unsaturated clayey materials (water being the wetting phase). It explains for the first time an important amount of literature data in the frequency range 1 mHz to 1 GHz in a very simple way.

## 2. Theory

**Figure 1:**
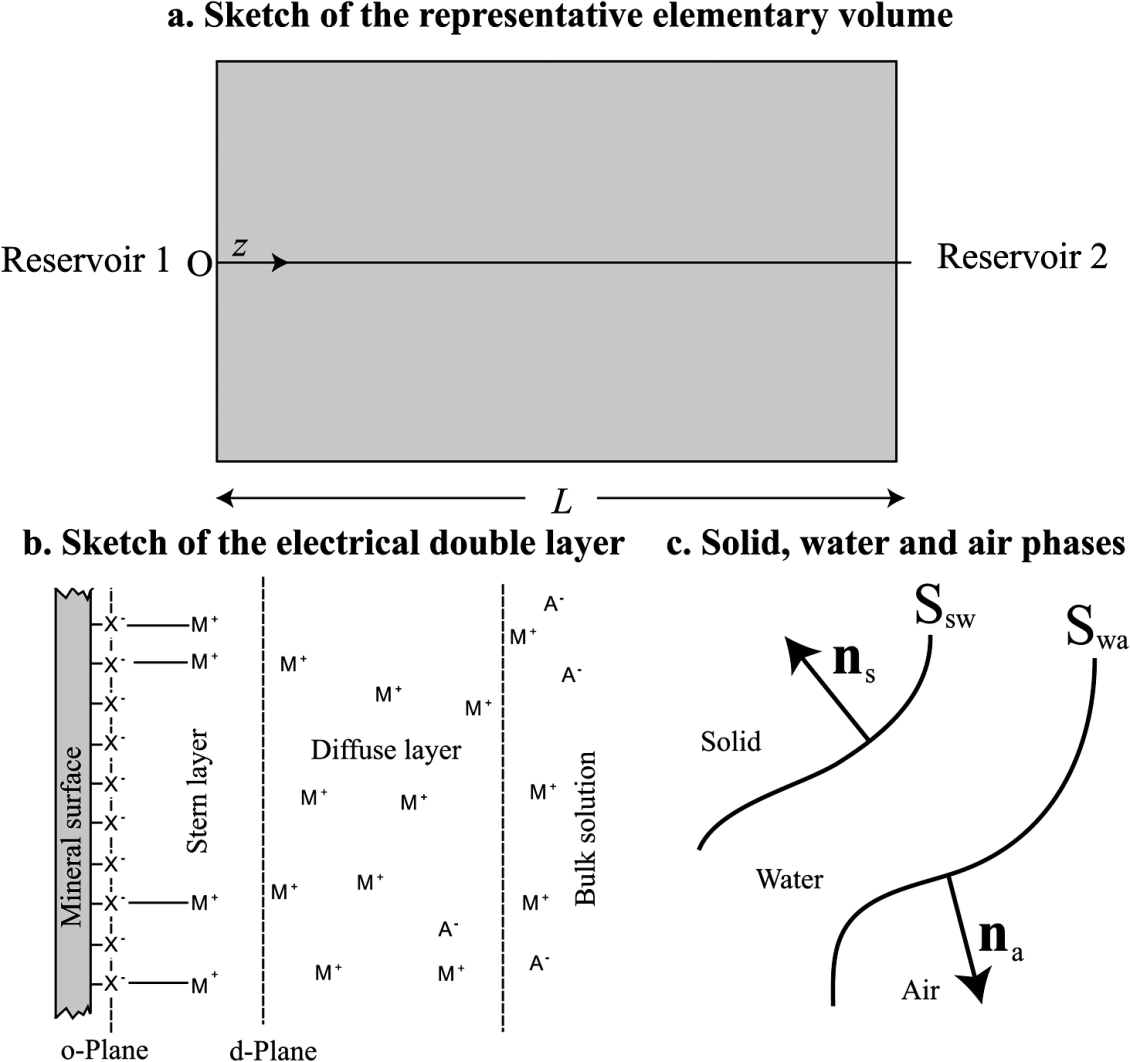
The REV. (a) Sketch of the REV comprised between two reservoirs. (b) Sketch of the electrical double layer at the solid water interface. The double layer includes the Stern layer and the diffuse layer. The charges of these two layers compensate the charge on the mineral surface (*o* plane). The *d* plane corresponds to the separation plane between the Stern and the diffuse layers. (c) Sketch of the solid-water and water-air interfaces.

**Figure 2:**
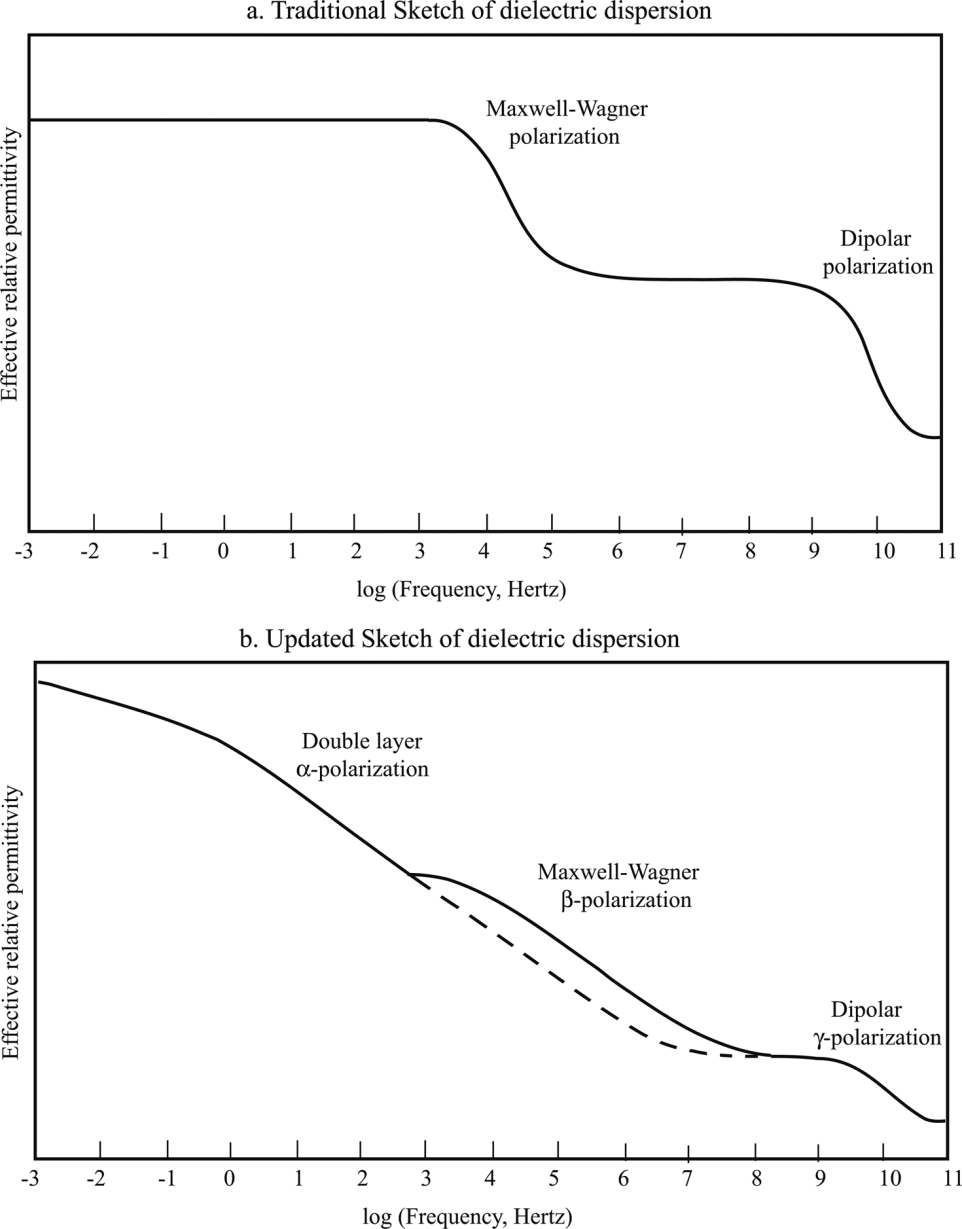
Dielectric dispersion for polarization in charged porous media. (a) Classical representation from *Poley et al*. [1978] and found in a number of papers in geophysics. (b) Representation of the three types of polarization discussed in the text. The *α* polarization corresponds to the polarization of the electrical double layer, the *β* polarization corresponds to the Maxwell-Wagner polarization, and the *γ* polarization concerns the polarization of the water molecules.

I consider an unsaturated porous material as shown in Figure 1a with an electrical double layer coating the surface of the grains (Figure 1b). In the classical literature in geophysics, it is customary to see a polarization sketch like the one shown in Figure 2a [e.g., *Hasted*, 1961]. In the frequency range 1 mHz–1 GHz, the apparent dielectric constant of a porous material is explained by two dissipation mechanisms only: the Maxwell Wagner polarization (called the β polarization in electrochemistry, see *Grosse* [2002]) and the polarization of the water molecules past the gigahertz frequency (called the γ polarization). That said, we know that in induced polarization, the polarization of the electrical double layer plays an important role below 10 kHz. This nondielectric dispersion mechanism is associated with the electromigration and accumulation/depletion of charge carriers at discontinuities in the migration pathways of the charge carriers. This mechanism is called the α polarization in electrochemistry, and its role is such that it may be the dominating mechanism of polarization for a very broad frequency range as discussed in this paper (see Figure 2b).

### 2.1. Effective Conductivity and Permittivity: Definitions

I first define the concepts of effective conductivity, effective permittivity, and effective quadrature conductivity. Ampère law is given by [e.g., *Vinegar and Waxman*, 1984],


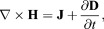
(1)

where **J** = σ* **E** denotes the conduction current density (in A m^−2^), 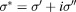 represents the complex conductivity, 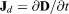 corresponds to the displacement current density (A m^−2^), **D** = *ε***E** represents the dielectric displacement (in C m^−2^), **H** denotes the auxiliary magnetic field (A m^−1^), and *ε* denotes the permittivity or dielectric constant (in F m^−1^) of the material. I consider an harmonic external electrical field 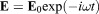 (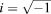 is the pure imaginary number, *f* is the frequency in Hz, ω = 2*πf* the angular frequency (in rad s^−1^), and **E**_0_ denotes a constant amplitude of the alternating electrical field). The total current density 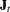 is the sum of **J** and 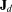. This yields 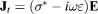 [*Vinegar and Waxman*, 1984]. This total current density can be written as,


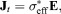
(2)

where 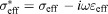 is the effective complex conductivity and 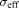 and 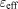 are real positive frequency-dependent scalars defined by


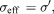
(3)


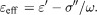
(4)

It is also possible to define an effective quadrature conductivity (in S m^−1^) that is directly related to the effective permittivity as,


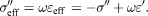
(5)

Note that in the conventions used above, 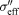 is positively defined while 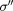 is negatively defined. Two phase angles can be defined. The former is determined from the complex conductivity 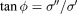 and the second from the effective parameters 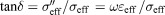 (note that 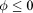 while 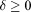).

### 2.2. Conductivity and Permittivity: A Volume-Averaging Approach

*Pride* [1994] developed a volume-averaging approach used to upscale the Nernst-Planck equation to the scale of a representative elementary volume (REV) of a porous rock. He was however concerned only by the saturated case and motivated by the modeling of the seismoelectric effect. We use this approach to upscale the Nernst-Planck equation to a porous material saturated by two immiscible fluid phases (water and air). The local current density 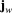 (in A m^−2^) occurring in the water phase is determined from the Nernst-Planck equation, which is written, in absence of macroscopic concentration gradients, as


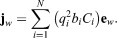
(6)

where 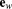 denotes the local electrical field (in V m^−1^), *N* denote the number of ionic species, *q*_i_ the charge of species *i*, and *C_i_* their concentration. The mobilities 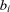 (in N s m^−1^) entering equation (6) are related to the classical ionic mobilities 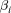 (in m^2^ s^−1^ V^−1^) by 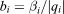 and to the ionic self-diffusion coefficients 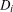 by 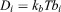, where *k_b_* denotes the Boltzmann constant (1.3807 × 10^−23^ J K^−1^), and *T* the absolute temperature (in K). There is also a current density associated with the grains, 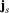 due to the electrical double layer coating the surface of the insulating grains. The boundary conditions at the interface 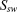 separating the solid phase and the pore space are


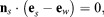
(7)


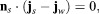
(8)

where 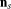 denotes the normal unit vector to 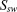 directed from the pore to the solid phase (Figure 1c). The pore space is filled with two immiscible fluids, the water and air phases (water is assumed to be the wetting phase). The boundary conditions at the interface 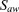 separating the nonwetting phase (air) and water are


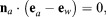
(9)


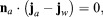
(10)

where 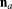 denotes the normal unit vector to 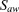 directed from the water to the nonwetting phase (Figure 1c). In the following, I will consider that the surface charge density at the interface between water and the nonwetting phase can be neglected. The reason to neglect this contribution is because there is still a gap of knowledge in describing the conductivity and polarization of this interface, which is, however, described by an electrical double layer [*Leroy et al*., 2012] and therefore likely characterized by surface conduction and polarization.

The microscopic equations given above are now averaged at the scale of an REV. The REV corresponds to the rock volume located in between two large-parallel circular disks of area *A* separated by the distance *L*. This is typically the case of a jacketed cylindrical sample in the laboratory. We assume that there is a macroscopic electrical potential difference 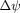. When this potential difference is divided by *L*, one obtains the equivalent macroscopic field perpendicular to the end faces of the REV. We note **z** the unit vector normal to the end faces (Figure 1a). For example, for the electrical field at the pore or grain scale, we have 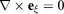, so the electrical fields 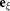 can be derived from scalar electrical potentials 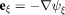 where the index *ξ* defined the phase (*w* for water, *s* for solid, *a* for air). The macroscopic electrical field is written as


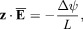
(11)

where 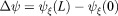 denotes the difference of electrical potential between the two reservoirs (Figure 1a). In the pore water phase, the fundamental Laplace problem is defined by


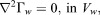
(12)


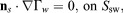
(13)


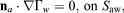
(14)


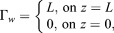
(15)

where *S_sw_* and *S_aw_* denote the solid-water and air-water interfaces, respectively (Figure 1c), and *V_w_* denotes the water phase volume. The electrical potential in the water phase can be written as 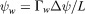 and the local electrical field by 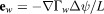. A similar boundary-value problem of the normalized effective potential 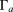 can be defined in the nonwetting phase (*V_a_* denotes the air phase),


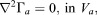
(16)


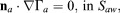
(17)


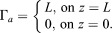
(18)

The volume average of a quantity 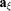 (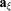 is a scalar, a vector, or possibly a tensor) is


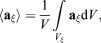
(19)

where 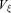 is the volume of the *ξ* phase within the REV. Slattery's classical theorem states [*Slattery*, 1967],


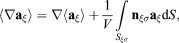
(20)

(no summation on the indices) where 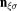 represents the unit outwardly directed normal vector for the 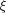 phase and 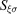 represents the interfacial area contained within the averaging volume. Applying this to the water, solid, and air phases and using the unit vectors defined in Figure 1c, I obtain,


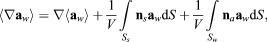
(21)


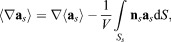
(22)


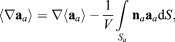
(23)

where d*S* is an infinitesimal surface volume element. The volumetric phase average 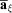 and the total volumetric average 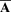 are rerelated to each other by,


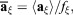
(24)


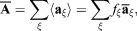
(25)

where 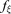 denotes the volumetric fraction of phase *ξ* (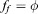, *ϕ* denotes the connected porosity, and 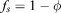 is the solidity, 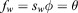 is the volume fraction of water, also called the water content in hydrogeology, and 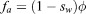 denotes the volume fraction of the nonwetting phase). Therefore, we have,


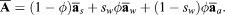
(26)

We first define the electrical conductivity of the pore water, which is given by,


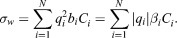
(27)

From equation (26), the average conduction current density is given by


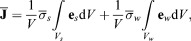
(28)


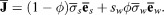
(29)

The tortuosity of the water phase 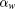 is defined by


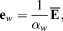
(30)


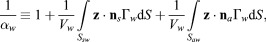
(31)

where 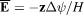 denotes the macroscopic electrical field. From equations (25) and (26), the macroscopic electrical field is also given by


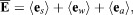
(32)


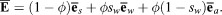
(33)

The phase average of the electrical field 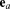 can be also related to the macroscopic electrical field via the tortuosity of the nonwetting phase 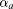,


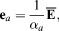
(34)


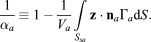
(35)

I need now to find a relationship between the tortuosity of the pore space *α* (related to the definition of the electrical formation factor *F* at saturation by 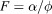) and the tortuosities of the wetting and nonwetting phases. If we note 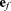 the local electrical field of the fluid phase (comprising the wetting plus nonwetting fluid phases), the phase average of 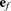 is related to the phase average of the electrical field in the wetting phase 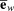 and to the phase average of the electrical field in the nonwetting phase 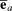 by


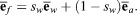
(36)

The electric field 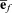 is related to the macroscopic field 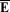 by,


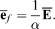
(37)

Combining equations (30), (34), (A36), and (37), I obtain after few algebraic manipulations the following relationship between the tortuosity of the pore space, the tortuosiy of the water phase, and the tortuosity of the air phase,


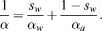
(38)

Therefore, the tortuosity of the pore space is equal to the harmonic average of the tortuosities of each phase weighted by their relative saturation. If we multiply equation (38) by the porosity, we obtain a relationship between the tortuosity of the air phase, the formation factor, and the tortuosity of the water phase,


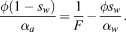
(39)

The electrical formation factor can in turn be related to the porosity by a power-law function [*Archie*, 1942],


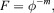
(40)

called the first Archie's law, where *m* is called the cementation exponent (*m* ≥ 1 is a strict bound and (1 ≤ *m* ≤ 3 for sandstones and sands according to experimental data, see *Revil et al*. [1998, Figure 5]). The conduction current density can be written as


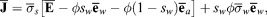
(41)


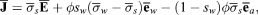
(42)


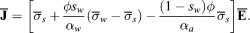
(43)

The effective electrical conductivity of the material 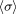 (in S m^−1^) is defined by the macroscopic Ohm's law,


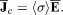
(44)

Combining equations (43) and (44) yields


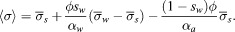
(45)

Note that in the case where there is no surface conductivity, we can connect equation (45) to the second Archie's law by


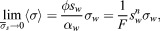
(46)

where *n* is called the second Archie's exponent in the literature. In the following, this second Archie's exponent in the expression of the electrical conductivity is introduced by using the following change of variables,


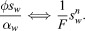
(47)

From equation (39), I can also use the following change of variables,


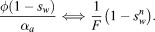
(48)

Using equations (47) and (48) into equation (45), I obtained the following expression for the electrical conductivity of the porous medium,


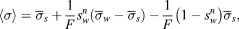
(49)


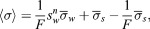
(50)


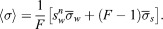
(51)

In the following, I remove the bar on the pore water and surface conductivity terms and drop the average symbol for the electrical conductivity to keep the notations as simple as possible. The same chain of algebraic manipulations could be applied also to complex conductivity and therefore I can write the following expression of the complex conductivity of the clayey material, σ*, as a function of the conductivity of the pore water 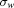 and the complex conductivity of the solid phase 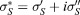 (complex because of the polarization the electrical double-layer coating the grains),


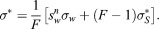
(52)

Note that the pore fluid conduction and surface conduction add in parallel in this model, and therefore the electrical conductivity of the porous material is a linear function of the conductivity of the pore fluid. This is expected as the volume-averaging approach treats the solid and fluid phases symmetrically, and they are both assumed to be continuous through the REV. As discussed in Appendix A, the situation is different when a differential effective medium approach is used. When we are dealing with granular media, the solid phase is discontinuous and grains are immersed in the background pore water. In this case, the differential effective medium theory yields a nonlinear relationship between the electrical conductivity of the porous material and the conductivity of the pore fluid (see for instance, *Revil et al*. [1998] and *Revil* [1999]). Sands and sandstones are somewhere in between these two types of models as the diffuse layer is continuous (overlapping from grain-to-grain) while the Stern layer is discontinuous. In addition, it follows that the diffuse layer does not polarize while the Stern layer polarizes and that the Stern layer cannot contribute to the DC electrical conductivity.

Using the displacement current density rather than the electromigration current density, an equation similar to equation (52) can be obtained for the high-frequency dielectric constant using the same chain of algebraic manipulations,


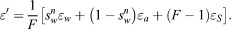
(53)

Now coming back to equation (52), I need to express the low-frequency conductivity of the solid phase corresponding to an insulating solid phase coated by a conductive and polarizable electrical double layer as a function of the surface charge density, the mobility of the counterions, and the tortuosity of the electromigration pathways along the surface of the grains. I assume that at low frequency, only the conduction of the electrical diffuse layer participates to the surface conductivity. Indeed, the grains are in contact to each other (a very important distinction with colloidal suspensions) and the diffuse layer is always above a percolation threshold. This assumption has been checked experimentally by *Vaudelet et al*. [2011a, 2011b].

The electrical conductivity of the grains is given by the mobility of the counterions (corrected by the tortuosity around the grains) times the concentration of the charge carriers times their charge. This yields,


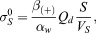
(54)

where *S* denotes the surface area of the grains (in m^2^), *Q_d_* is the surface charge density of the diffuse layer (in C m^−2^), and *V_S_* is the volume of the grains (in m^3^). Note that the mobility of the charge carriers is corrected for the tortuosity of the electromigration of the counterions along the surface of the grains in the water phase (the counterions are not allowed to pass through the grains that are insulating). The fraction of the volumetric charge density associated with the diffuse layer is written as


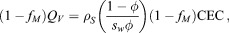
(55)

where 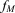 (dimensionless) denotes the fraction of counterions in the Stern layer (the counterions compensating the negative charge of the mineral surface are distributed between the diffuse layer and the Stern later, see *Revil* [2012] and references therein). Consequently, 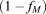 denotes the fraction of counterions in the diffuse layer. The volumetric charge density 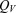 (in C m^−3^) denotes the total charge density of the double-layer per unit volume of the pore water (including the Stern and diffuse layers taken together). The relationship between 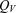 and its value at saturation (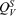 at 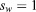) is 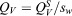. We also have


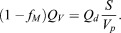
(56)

Therefore, the surface charge density can be expressed in terms of the CEC by


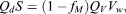
(57)


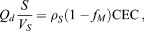
(58)

where equation (55) has been used. Therefore, the real (in phase) low-frequency component of the solid phase conductivity is given by,


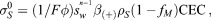
(59)

where I used the following change of variables 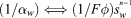 resulting from equation (47). I replace below the exponent (*n* − 1) in the expression of the complex surface conductivity by the exponent *p* because the true tortuosity that should be used is the one associated with pathways along the grains, not strictly speaking, the tortosity of the water phase adjacent to the grains (I will show however in section 7 that the approximation *p* ≍ *n* − 1 is a good one). This approach is consistent with the one derived by *Vinegar and Waxman* [1984] and *Slater and Glaser* [2003]. The low-frequency (in phase) conductivity is given by


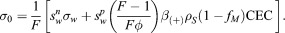
(60)

A similar approach can be followed for the high-frequency electrical conductivity, for which now both the Stern and diffuse layers contribute to the overall surface conductivity [*Revil*, 2012; *Revil et al*., 2012a]. Following the same approach as used to derive equation (60), this yields


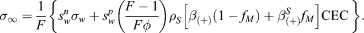
(61)

where 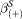 denotes the mobility of the counterions in the Stern layer (as expected 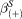 ≪ 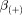, see *Revil* [2012] for clays and *Revil et al*. [2012a] argued for 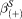 = 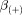 in silica).

An important parameter in the description of frequency-domain induced polarization, but more importantly in the description of time domain induced polarization, is the normalized chargeability. In a time-domain induced polarization experiment, the chargeability *M* characterizes how the decay of the secondary voltages when the primary current is shut down. This chargeability can be normalized to obtain the normalized chargeability 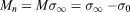, and therefore from equations (60) and (61), the normalized chargeability is given by


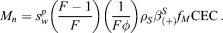
(62)

We can express also the conductivity terms in term of the volumetric charge density 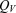, which yields,


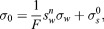
(63)


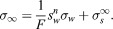
(64)

in which we have entered the term 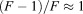 in the expressions of the surface conductivities. In these relationships, the low-frequency and high-frequency surface conductivity terms are expressed as


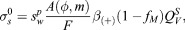
(65)


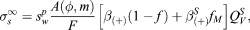
(66)

and the parameter 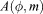 is given by


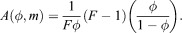
(67)

Note that the ratio of the surface conductivity to the pore water conductivity is often called the Dukhin number (*Du*) in the literature. The expression for 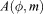 contrasts with the high salinity conductivity model developed recently by *Revil* [2012], which yields,


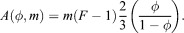
(68)

For spherical grains, 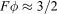 (more precisely π/2 for the ions to move around a perfect spherical grain) and therefore the presently derived expression of 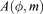 is


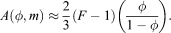
(69)

The difference between equations (67) and (68) is therefore really in the factor *m* in the expression derived from the effective medium theory. Note for spherical grain (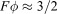) and at high porosity, the formation factor is given by


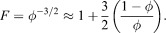
(70)

Equations (69) and (70) yields


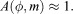
(71)

With this approximation and using *p* = *n* − 1, the high-frequency and low-frequency conductivities are given exactly by the *Waxman and Smits* [1968] equation (see also *Waxman and Thomas* [1974] for the quadrature conductivity):


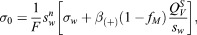
(72)


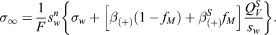
(73)

The present analysis therefore provides a theoretical foundation to the *Waxman and Smits* [1968] model in which their mobility *B* is related to the mobility 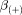 by 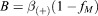.

In the high-porosity limit discussed previously, the normalized chargeability is given as


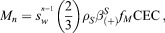
(74)

which is exactly equation (43) of *Revil* [2012] for the quadrature conductivity obtained through the differential effective medium approach. Up to this point, I have not discussed the frequency dependence of the electrical conductivity. This will be done in the next section. Also, rather than writing the surface conductivity and the normalized chargeability as a function of the CEC, we can use the relationship developed by *Revil* [2012] to express the parameters as a function of the specific surface area. The relationship between the CEC (in C kg^−1^) and the specific surface area *S_Sp_* (in m^2^ kg^−1^) is 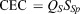, where 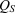 denotes the mean charge density for clay minerals (typically, two elementary charges per nm^2^, i.e., 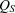 = 0.32 C m^−2^, see *Revil* [2012]). Note that 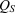 and 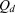 are related to each other by 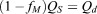.

### 2.3. Frequency Dependence of the Effective Conductivity and Permittivity

**Figure 3:**
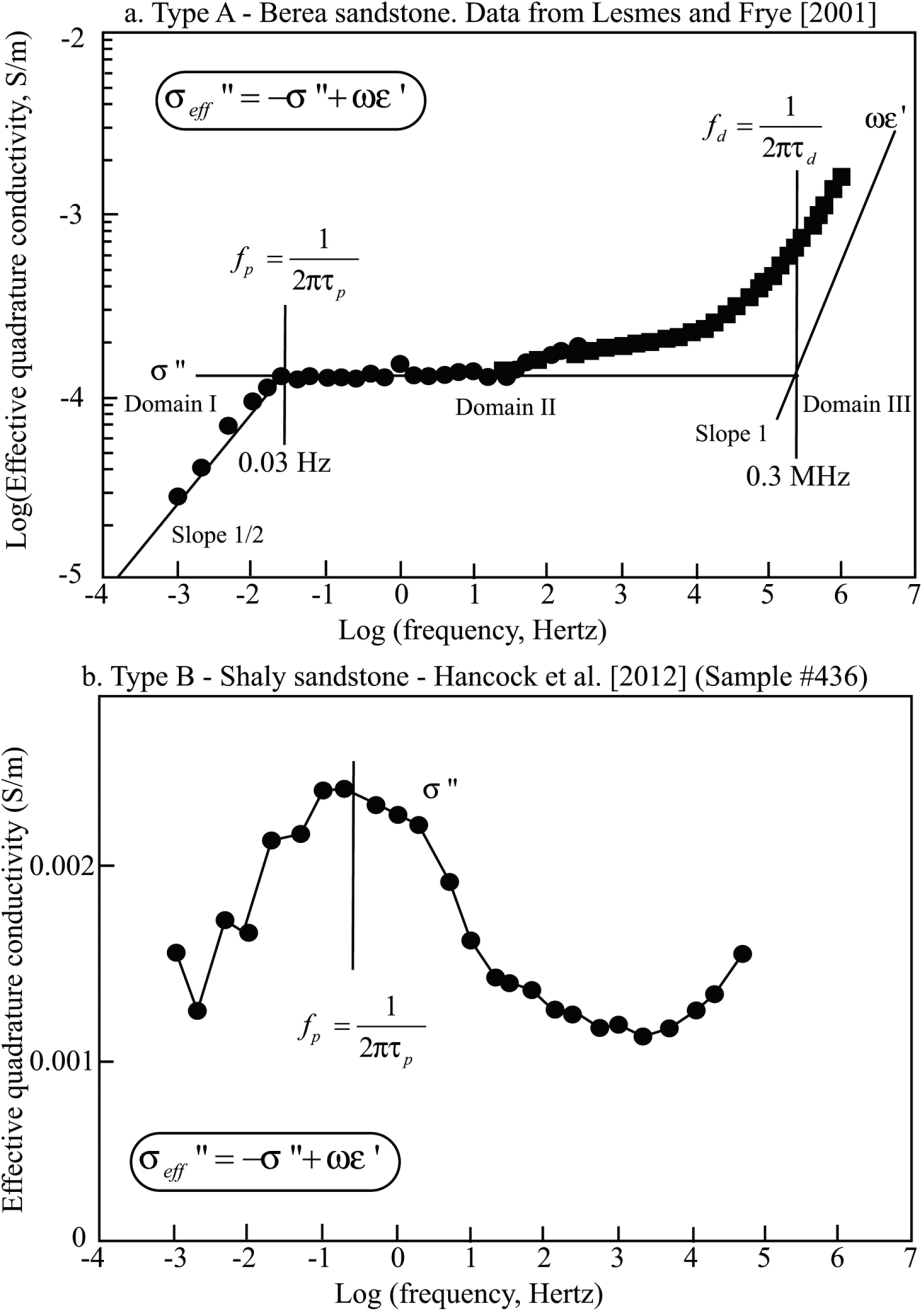
Effective quadrature conductivity versus frequency. (a) Type A. Berea sandstone (data from *Lesmes and Frye* [2001], pH 8, 10^−1^ M NaCl solution). The data are characterized by three components: a minimum critical frequency from which the magnitude of the quadrature conductivity is going down when the frequency decreases, a plateau (over at least four decades in frequency), and a high-frequency domain in which there is an increase of the magnitude of the apparent quadrature conductivity. Filled circles: measurements with four electrodes, filled squares, measurements with two electrodes. (b) Type B. shaly conglomerate (this study, pH 7, 10^−1^ M NaCl solution).

Figure 3 shows the effective quadrature conductivity spectrum for a Berea sandstone (data from *Lesmes and Frye* [2001]), which is a clayey sandstone with a very small amount of clay minerals. The properties of the Berea sandstone are summarized in Tables 1 and 2. I call Type A, clayey materials showing the type of quadrature conductivity spectra shown in Figure 3a. This type of quadrature conductivity distribution exhibits three distinct regions: (i) Domain I is defined by frequencies such as 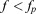 (≍ 0.03 Hz for the Berea sandstone data shown in Figure 3a). In this region, the effective quadrature conductivity decreases with the frequency (with 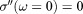). Domain II is defined by 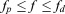 (≍ 0.3 MHz in Figure 3a). In this domain, the quadrature conductivity reaches a plateau. Domain III is defined by 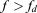. In this domain, the effective quadrature conductivity increases with the frequency and is controlled by the permittivity of the material. I first provide some expressions for the two critical frequencies and then I will provide expressions for each domain.

In Appendix B, I show that a characteristic relaxation time of a clayey material can be determined by the following equations


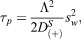
(75)


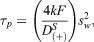
(76)

where 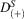 denotes the diffusion coefficient of the counterions (in m^2^ s^−1^), *k* denotes the permeability (in m^2^), and Λ is a characteristic pore throat size that can be derived from the permeability and the formation factor (see for instance, Table 2 for the Berea sandstone and a definition of this parameter in Appendix B). The diffusion coefficient is related to the mobility of the counterions in the Stern layer, 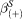, by the Nernst-Einstein relationship 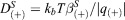, where 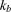 is the Boltzmann constant (1.3807 × 10^−23^ J K^−1^), *T* is the absolute temperature (in K), and 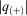 is the absolute value of the charge of the counterions in the Stern layer. Using the database of *Vinegar and Waxman* [1984], *Revil* [2012] found 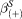
(Na^+^, 25°C) = 1.5 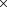 10^−10^ m^2^s^−1^V^−1^. This yields the diffusion coefficient of the counterions 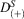
(Na^+^, 25°C) = 3.8 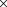 10^−12^ m^2^ s^−1^ for clays. For fully water-saturated materials, the lower critical frequency at which the effective quadrature conductivity becomes constant is given by


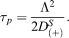
(77)

Taking Λ = 6 µm and 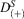
(Na^+^, 25°C) = 3.8 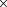 10^−12^ m^2^ s^−1^, the critical frequency associated with the previous relaxation time is


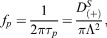
(78)


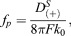
(79)

and I obtain 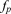 = 0.034 Hertz in excellent agreement with the data shown in Figure 3a. Note the measurement of this relaxation frequency can be used to estimate therefore the permeability of the material using equation (79) (this idea will be explored below in section 6).

I follow here closely *Vinegar and Waxman* [1984] in estimating the highest frequency at which the Stern layer polarization can play a role. The shortest possible spatial variation between charged and uncharged zones is the average distance between the surface sites on the clay minerals. I have mentioned that the surface charge density on the surface of clay minerals is in average 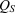 = 0.32 C m^−2^ (see *Revil* [2012]). So the average distance between the charged sites is 1.8 nm. Using this distance, equation (78) yields a critical frequency of 0.3 MHz.

The second characteristic frequency comes from the transition between the low-frequency polarization of the electrical double layer and the high-frequency dielectric effect in the overall polarization of the material. It is therefore defined by


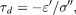
(80)

where 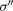 and 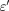 denotes the quadrature conductivity of the plateau and the high-frequency dielectric constant. From equation (53), the high-frequency dielectric constant is given (for saturated conditions) by


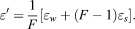
(81)

Taking 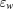 = 80 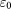, 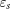 = 5.9 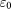, and *F* = 18 for the Berea sandstone, we get 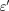 = 10.0 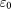. The second critical frequency is therefore given by


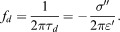
(82)

Using the value of the quadrature conductivity for the Berea sandstone, 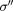 = −1.5×10^−4^ S m^−1^ (see Figure 3), and 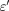 = 10.0 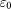 yields 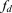 = 0.3 MHz (see Figure 3a). Above this frequency, the polarization response of the material is dominated by the dielectric polarization of the material (see Figure 3a). Note also if the quadrature conductivity is on the order of −1 × 10^−5^ S m^−1^, the critical frequency 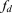 can be as low as 10 kHz.

I now describe the quadrature conductivity response below the critical frequency *f_p_* for Type A materials, which are exhibiting a clear plateau in their polarization spectra. This plateau is likely resulting from a broad distribution of polarization length scales and therefore from the self-similarity of the pore space for a broad range of scales (this idea was expressed by *Vinegar and Waxman* [1984] and is consistent with the modeling performed by *Cosenza et al*. [2008] and observations by *Hyslip and Vallejo* [1997]).

**Figure 4:**
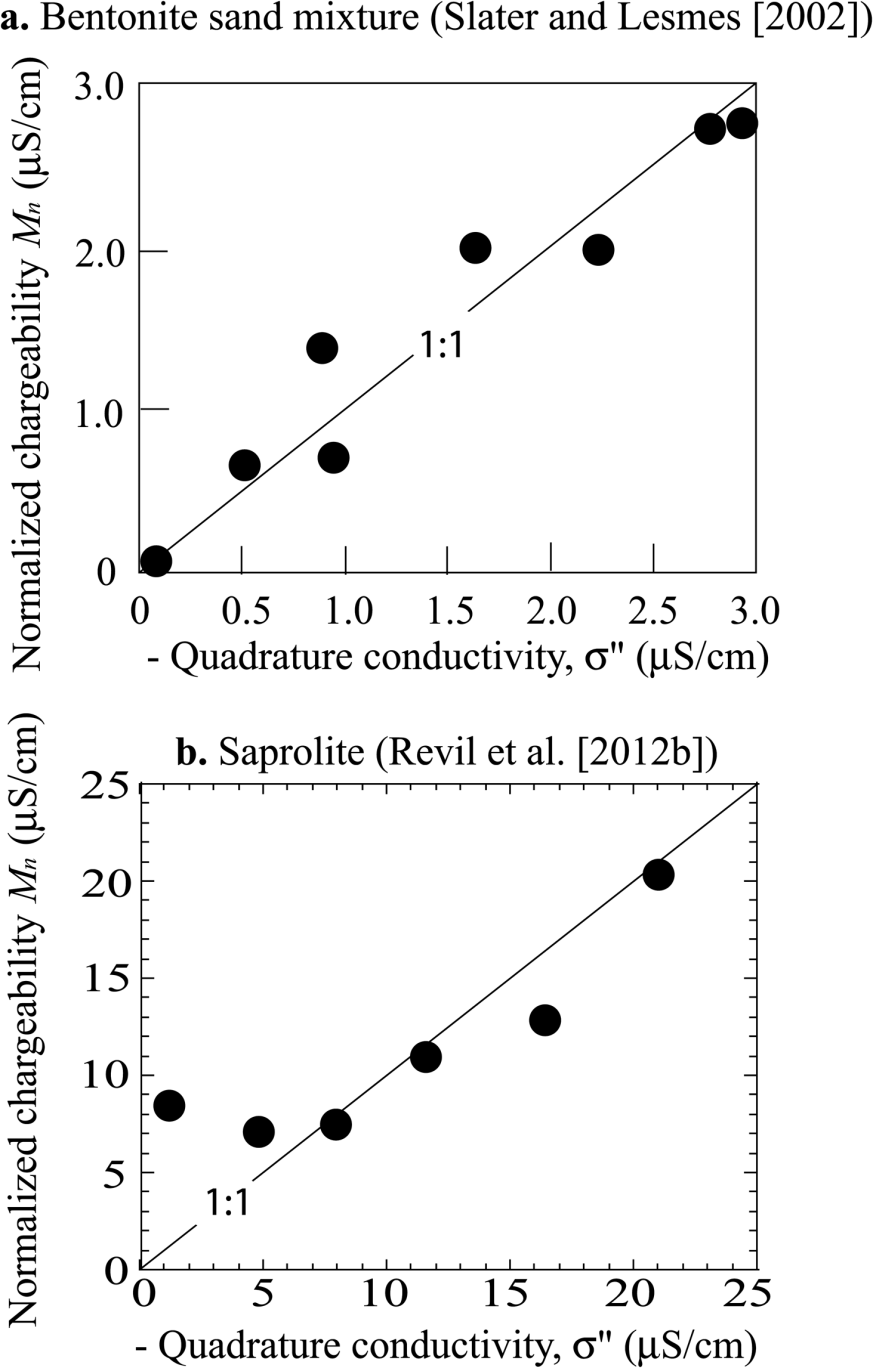
Relationship between the normalized chargeability and the quadrature conductivity. (a) Eight bentonite sand mixtures (*R* = 0.97). Data from *Slater and Lesmes* [2002]. (b) One saprolite core sample (S16, see *Revil et al*. [2012b]). The normalized chargeability is determined from the high and low asymptotic values of the (in-phase) electrical conductivity. The discrepancy at low quadrature conductivity may be due to the higher uncertainty in extrapolating the high-frequency and low-frequency limits for the electrical conductivity.

For clay minerals, the kinetics of sorption/desorption of cations in the Stern layer is pretty fast (few seconds according to *Pohlmeier and Ilic* [1998]). Therefore at low frequencies (typically < 1 Hertz), the kinetics of sorption/desorption may influence the polarization of the electrical double layer. *Wong* [1979] provided a general model describing the polarization of metallic particles (grains) exchanging charge carriers with the surrounding pore water. In his case, the charge carriers are redox active species (electron donors and electron acceptors) and the particle conductive to electrons. His model is however general to work also with insulating grains coated by an electrical double layer. *Wong* [1979] showed that his rather complex model can be represented with a Warburg impedance model. The Warburg model implies that at low frequencies (*f* < *f_p_*), the quadrature conductivity decreases as the inverse of the root mean square of the frequency. For type A materials exhibiting a plateau for the quadrature conductivity (see Figure 3a), the normalized chargeability is also equal to the quadrature conductivity (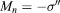). Note that *Lesmes and Frye* [2001] and *Slater and Lesmes* [2002] attempted a semitheoretical explanation for the equivalence between normalized chargeability and imaginary conductivity. This equivalence is very well illustrated by the data of *Slater and Lesmes* [2002] and *Revil et al*. [2012b] (see Figure 4).

**Figure 5:**
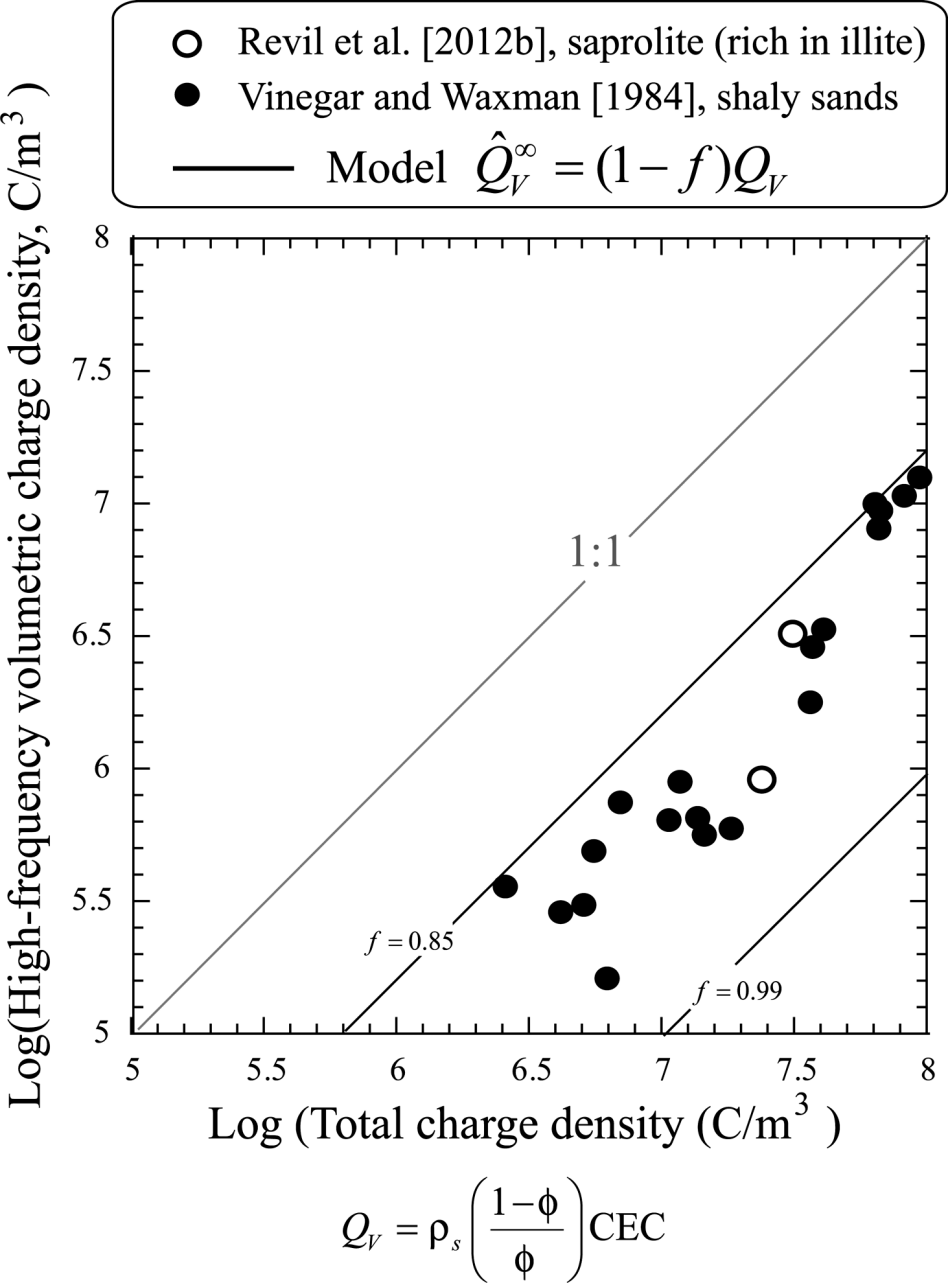
High-frequency volumetric charge density versus the total charge density. The high-frequency volumetric charge density is determined from electrical conductivity measurements at various salinities (from the surface conductivity and the formation factor), while the total charge density is determined from the porosity and the cation exchange capacity.

We therefore propose the following model in which the frequency dependence is explicit:


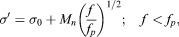
(83)


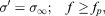
(84)


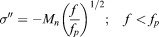
(85)


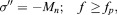
(86)

and where 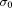 and 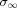 are given by equations (63) and (64), respectively, and the normalized chargeability 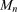 by equation (74). This (empirical) model shows that the quadrature conductivity is going to zero at zero frequency [*Fuller and Ward*, 1970; *Vinegar and Waxman*, 1984]. Therefore, from equations (10) to (15), the effective conductivity and the effective dielectric constant are expressed as


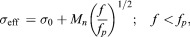
(87)


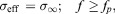
(88)


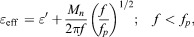
(89)


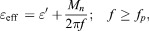
(90)

where the high-frequency dielectric constant 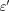 is given by equation (53).

Type B spectra are those showing a clear peak in the low-frequency part of the spectrum (see Figure 3b for which, however, the data are plotted in a linear scale in quadrature conductivity). In this case, we expect a Warburg-type model to represent the low-frequency complex conductivity [see *Wong*, 1979]. Because the Warburg model is a special case of the Cole-Cole model (Cole-Cole exponent *c* = 1/2), I propose the following Cole-Cole model to represent the Type B spectra,


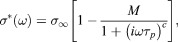
(91)


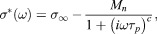
(92)

where 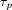 = 1/(2*πf_p_*) denotes the main relaxation time (in s) *f_p_* denotes the associated critical frequency, and *c* denotes the Cole-Cole exponent (the Warburg model corresponds to the case where *c* = 0.5, and I expect more generally that 0 ≤ *c* ≤ 0.5), and *M* denotes the chargeability (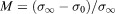). The real and imaginary parts of the complex conductivity of the Cole-Cole model are given by [*Cole and Cole*, 1941],


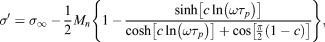
(93)


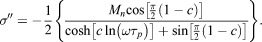
(94)

Therefore, from equations (10) to (15), the effective conductivity is given by 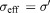 and the effective dielectric constant are expressed as


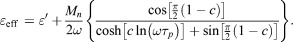
(95)

As mentioned earlier, I expect to have the Cole-Cole exponent *c* in the range 0 (flat response) to 0.5 (pure Warburg response). In the following, I will focus more on Type A spectra [see *Vinegar and Waxman*, 1984; *Slater and Lesmes*, 2002] rather than on Type B [*Scott and Barker*, 2003; *Binley et al*., 2005].

## 3. Effective Conductivity: Theory Versus Data

### 3.1. Determination of the Fraction of Counterions in the Stern Layer

The first test concerns the inference of the fraction of counterions in the Stern layer, *f_M_*. I use equations (63) and (65) with equation (69) for the parameter 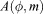 to reinterpret the electrical (in phase) conductivity data from *Vinegar and Waxman* [1984] on shaly sands. The results are reported in Table 3 and the values of *f_M_* determined from the porosity, the formation factor, and the surface conductivity are shown as a function of the excess of charge per unit pore volume in Figure 5. The high-frequency excess charge density 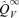 is estimated from the surface conductivity and the formation factor by


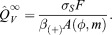
(96)

We see that a good average value for *f_M_* is close to 0.9 [see also *Revil*, 2012]. This means that approximately 90% of the counterions are, in average, located in the Stern layer in agreement with triple layer models (see discussion in *Revil* [2012]).

### 3.2. Salinity Dependence of the In-Phase Electrical Conductivity

**Figure 6:**
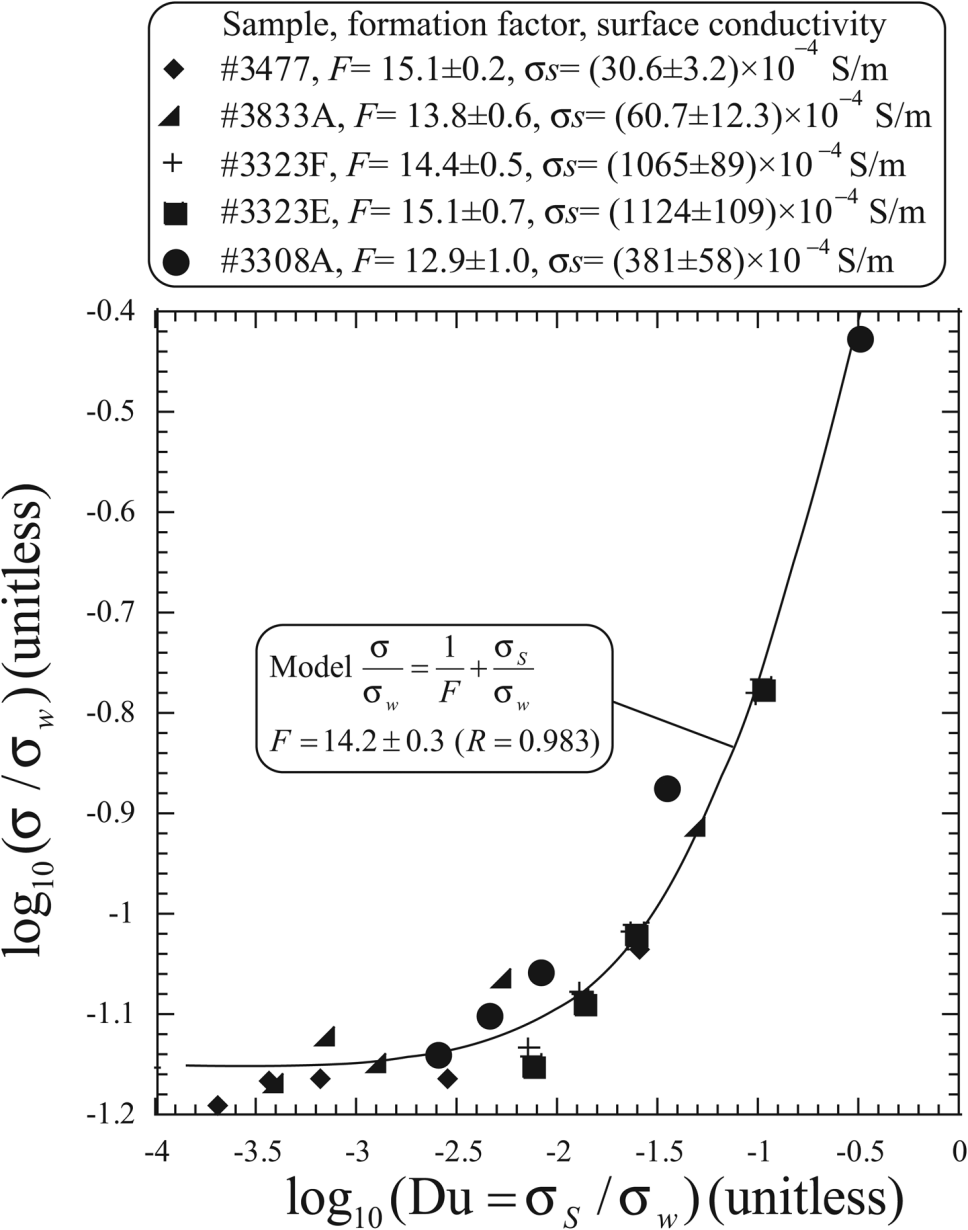
Pore water conductivity dependence of the conductivity of shaly sands (data from *Vinegar and Waxman* [1984]). I use here a collection of five samples with more or less the same formation factor but different CEC and therefore surface conductivities. According to the model developed in the main text, the conductivity data should follow the same trend, which is the case. The parameter *Du* denotes the Dukhin number, i.e., the ratio of surface to pore water conductivity.

In Figure 6, I investigate if the linear conductivity model described in the present modeling approach describes well the conductivity data of shaly sands. I use five samples from the *Vinegar and Waxman* [1984] database with roughly the same formation factor (in the range 12.9 to 15.1). According to the model, the conductivity data normalized by the conductivity of the pore water should follow a unique trend when plotted as a function of the Dukhin number defined as the ratio of surface to pore water conductivity. This is the case in Figure 6, indicating that the linear conductivity model described in the present paper works well in predicting the pore water conductivity dependence of the electrical conductivity of clayey materials. This is probably because of the continuity of the diffuse layer from grain-to-grain as mentioned in section 2.

## 4. Low-Frequency Effective Permittivity

**Figure 7:**
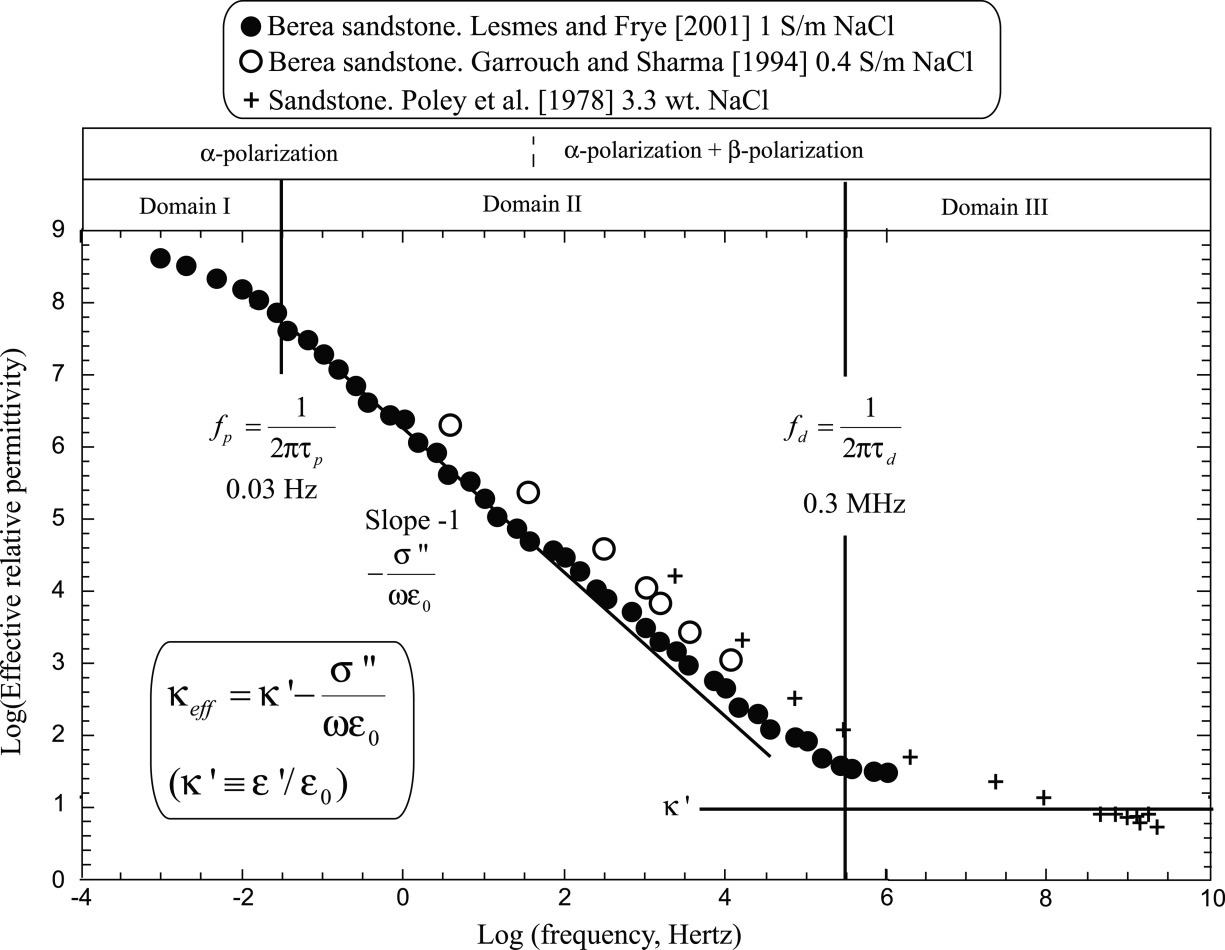
Effective relative permittivity versus frequency for the Berea sandstone (data from *Lesmes and Frye* [2001], pH 8, 10^−1^ M NaCl solution, *Garrouch and Sharma* [1994], 2000 ppm NaCl, and *Poley et al*. [1978], sandstone porosity 0.145, salinity NaCl 3.3% wt.). These data are the same than shown in Figure 3a but plotted in terms of the effective permittivity rather than in term of effective quadrature conductivity. In the geophysical literature, it is very frequent that the *α* polarization in Domain II is (wrongly) assumed to be a Maxwell-Wagner polarization.

In this section, I test five predictions of the model either for the effective quadrature conductivity or the low-frequency effective dielectric constant (these two parameters are related to each other by equation (5) in section 2.1). Figure 7 shows a typical effective relative permittivity spectrum. In this section, we are interested to test the model for frequency much below the critical frequency 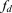.

### 4.1. Quadrature Conductivity: Influence of the Specific Surface Area

**Figure 8:**
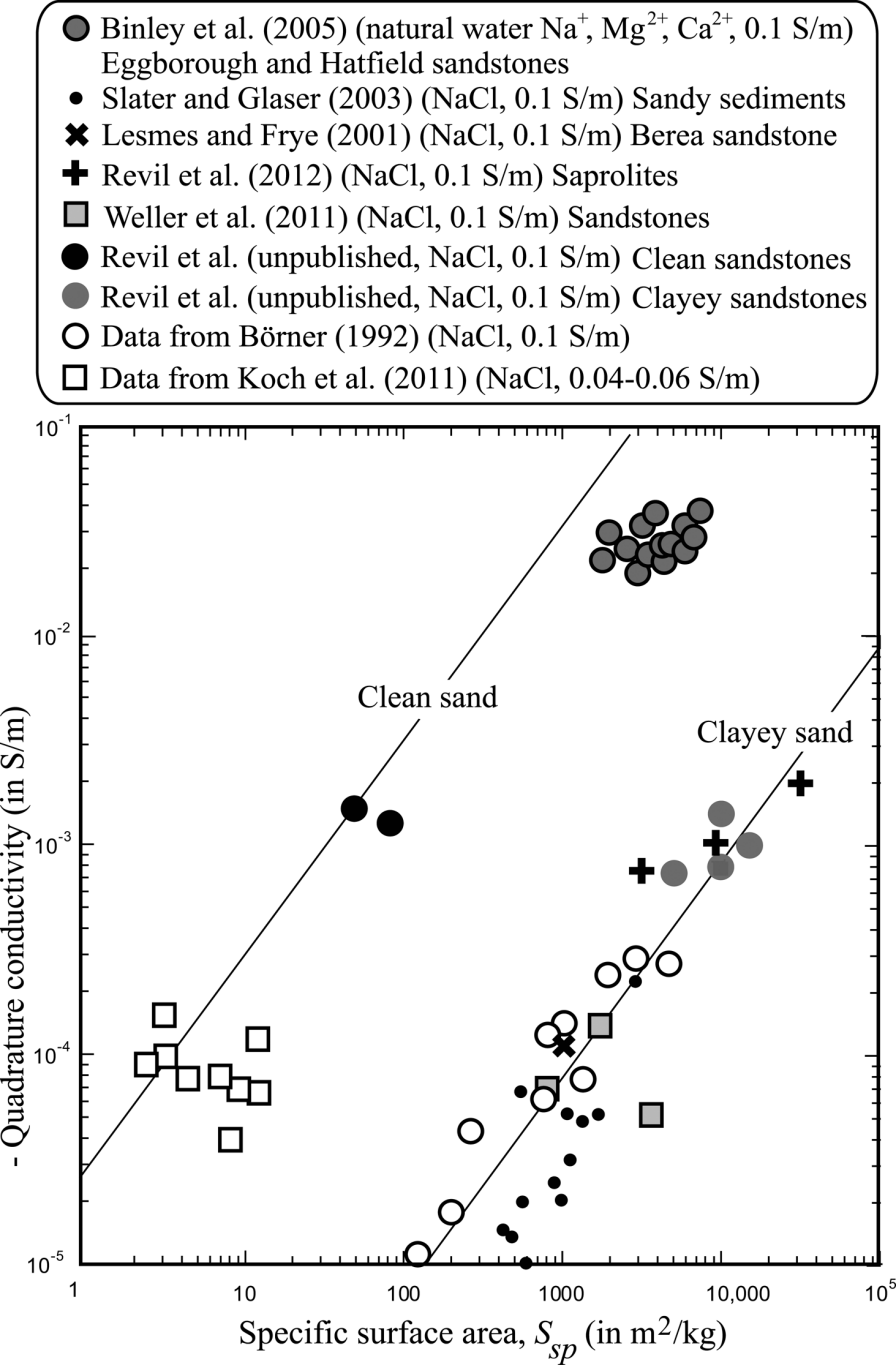
Influence of the specific surface area *S_Sp_* upon the quadrature conductivity. Trend determined for clayey sands from the model developed by *Revil* [2012] at 0.1 S m^−1^ NaCl. Data from *Slater and Glaser* [2003], *Weller et al*. [2011], *Börner* [1992], *Koch et al*. [2011], *Revil et al*. [2012b], and *Lesmes and Frye* [2001]. Measurements reported at 10 Hertz for the clayey materials, at 1.4 Hz for the data from *Binley et al*. [2005].

From equations (74) and (86) and for Type A spectra, the quadrature conductivity of the plateau at saturation of the water phase is given as a function of the CEC or the specific surface area by


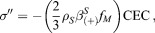
(97)


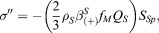
(98)

where we have used the relationship between the CEC and the specific surface area 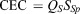 [see *Revil*, 2012]. For clayey sands, taking 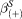
(Na^+^) = 1.5 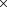 10^−10^ m^2^ s^−1^ V^−1^ at 25°C, *f* = 0.90, *Q_S_* = 0.32 C m^−2^, and *ρ_g_* = 2650 kg m^−3^, I obtain 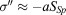 with *a* = 7.6 × 10^−8^ S kg m^−3^ [*Revil*, 2012]. For clean sands and sandstones, if we use 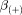
(Na^+^) = 5.2 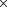 10^−8^ m^2^ s^−1^ V^−1^, *f* = 0.50, *Q_S_* = 0.64 C m^−2^, *ρ_g_* = 2650 kg m^−3^, I obtain *a* = 2.9×10^−5^ S kg m^−3^ [*Revil et al*., 2012a]. These two linear trends are shown in Figure 8 together with data from the literature. For the data of *Binley et al*. [2005], I converted the *S*_por_ data into specific surface area data using 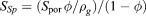 with a grain mass density of 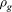 = 2650 kg m^−3^ and a mean porosity of 0.28. The model agrees with the data especially for clayey materials. For nonclayey materials, additional data points are needed. This demonstrates also the strong difference of the mobility of the counterions in the Stern layer for silica sands and for clays. This point may be, in turn, related to the density of counterions in the Stern layer of silica and aluminosilicates. In the case of silicate, only a small fraction of the active surface sites are locally associated with the sorption of the counterions in the Ster layer. For clay minerals, I have mentioned above that in average 90% of the charged surface sites are associated with counterions present in the Stern layer.

### 4.2. Effective Permittivity: Influence of the CEC and Surface Area

At low frequency, we can neglect the influence of the true dielectric constant in the effective permittivity. We first look for an expression of the effective relative permittivity as a function of the CEC and specific surface area at full saturation. We obtain


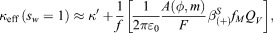
(99)


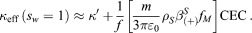
(100)

**Figure 9:**
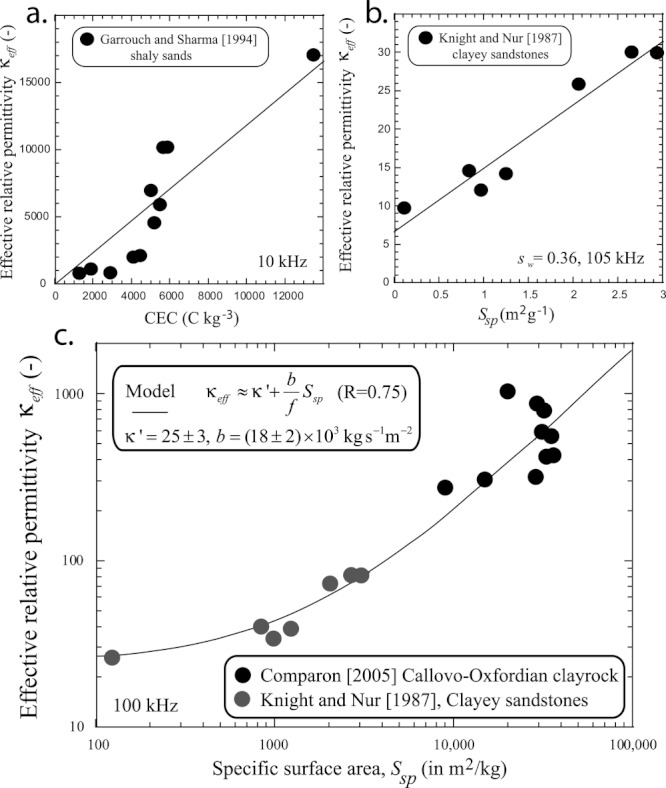
Influence of the CEC and specific surface area *S_Sp_* upon the effective relative permittivity. (a) At low frequencies, the relative effective permittivity is linearly correlated with the CEC in agreement with the model developed in the main text. (b) At low frequencies, the relative effective permittivity is also linearly correlated with the specific surface area *S_Sp_* (in m^2^ g^−1^) (data from *Knight and Nur* [1987], various sandstones including three Berea sandstones). (c). Measurements at 100 kHz. The line corresponds to the trend predicted by the model. Data from *Comparon* [2005] (100 kHz, clayrocks) and *Knight and Nur* [1987] (105 kHz, sandstones, corrected for the saturation by dividing with the saturation).

According to my model, the low-frequency effective relative permittivity is therefore proportional to the CEC. This is in agreement with the experimental results reported in Figures 9a and 9c. The slope of the trend shown in Figure 9a is 1.19±0.13 kg C^−1^. Taking *m* = 2, *f* = 10 kHz, ρ*_S_* = 2650 kg m^−3^, 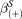 = 1.5 × 10^−10^ m^2^ V^−1^ s^−1^, *f*_M_ = 0.9, the predicted slope is 0.9 kg s^−1^ C^−1^, therefore, in excellent agreement with the observations. Equation (100) can be also expressed in terms of the specific surface area,


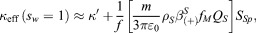
(101)

which can be written simply as,


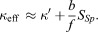
(102)

where *b* is a constant independent on the specific surface area. In Figure 9c, we report measurements of the relatively effective permittivity as a function of the specific surface area. A fit of these data with equation (102) yields 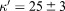 and 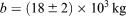 s^−1^ m^−2^.

### 4.3. Effective Permittivity: Influence of the Saturation

**Figure 10:**
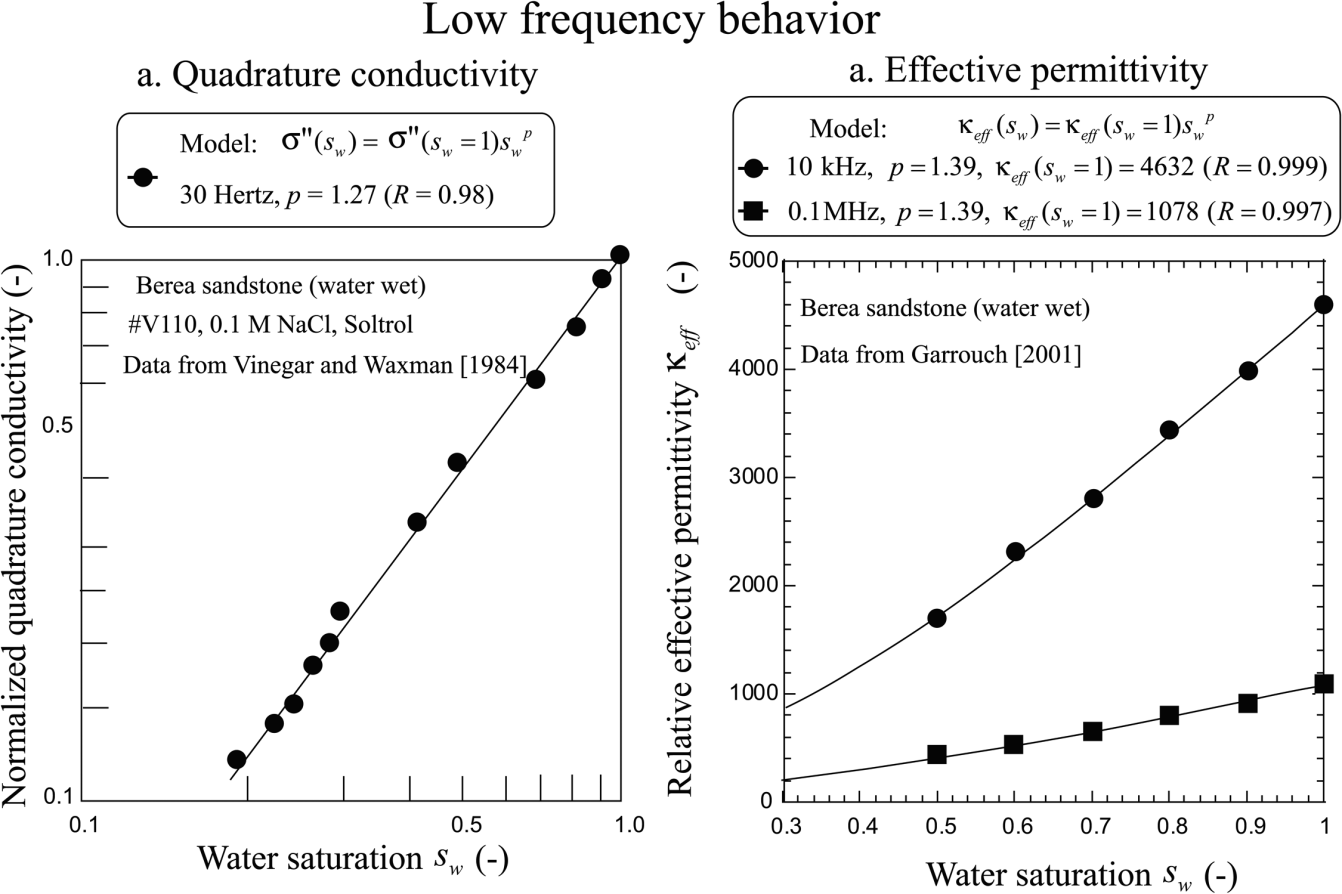
Saturation dependence of the quadrature conductivity and effective permittivity for the Berea sandstone. (a) Quadrature conductivity (data from *Vinegar and Waxman* [1984], 30 Hz, soltrol, 25°C). Soltrol is an isoparaffin solvent. (b) Relative effective permittivity of the Berea sandstone at two distinct frequencies (10 kHz and 100 kHz) showing that the permittivity follows a power-law function of the saturation (data from *Garrouch* [2001], the Berea sandstone is water-wet and saturated by *n*-decane and 10,000 ppm NaCl brine ∼2 S m^−1^, porosity 0.20). Note the consistency of the value of the *p* exponent. The plain lines correspond to the prediction of the model.

The third point to check is the dependence of the low-frequency effective relative permittivity with the saturation. We consider frequencies low enough so the true dielectric constant can be neglected. The saturation dependence of the effective relative permittivity is therefore given by


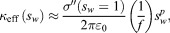
(103)

where *f* is the frequency (in Hertz) and 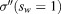 represents the value of the quadrature conductivity at saturation. We therefore see that the permittivity follows a power-law function with the saturation. This behavior is in very good agreement with the data of *Garrouch* [2001] (see Figure 10b). The saturation exponent *p* is equal to 1.4, fairly consistent with *p* = 1.3 resulting from the low-frequency quadrature conductivity measurements of *Vinegar and Waxman* [1984, Table 8] (see Figure 10a). *Vinegar and Waxman* [1984] reported that the saturation exponent *n* is in the range 2.1–2.2 for the Berea sandstone (see Table 1). This would yield *p* = *n* −1 ≍ 1.1−1.2 only slightly below the previous estimates (1.3–1.4).

**Table 1:** Properties of the Berea Sandstone (Clay Type: Kaolinite and Illite)

Property	Value
Archie exponent *m* (–)	1.78,^a^ 1.69,^b^ 1.85 ± 0.05^e^
Saturation exponent *n* (–)	1.83,^a^ 1.98 ± 0.23,^e^ 2.11 – 2.17^i^
Specific surface area *S_sp_* (m^2^ g^−1^)	0.74^c^ – 0.93^d^
Porosity *ϕ* (–)	0.20 ± 0.03^c^
Formation factor *F* (–)	18 to 26^b^
Grain density *ρ_S_* (kg m^−3^)	2660^i^
CEC (C kg^−3^)	256 C kg^−1^^h^
Excess charge density *Q_v_* (C m^−3^)	2.7 × 10^6^^j^ – 5.8 × 10^6^^i^

a Jun-Zhi and Lile [1990].

b From the raw data of *Lesmes and Frye* [2001], corrected for surface conductivity.

c *Lesmes and Frye* [2001] (one sample).

d *Zhan et al*. [2010].

e *Attia* [2005] (eight samples).

f *Aggour et al*. [1994].

g *Zhu and Toksöz* [2012]

h Using *CEC* = *Q_s_ S_sp_* with *Q_s_* = 0.32 C m^−2^ and *S_sp_* = 0.8 m^2^ g^−1^.

i *Vinegar and Waxman* [1984, Tables 7 and 10] (porosity 0.19, four samples).

j Using *Q_V_* = *ρ_S_* [(1 − *ϕ*)/*ϕ*] CEC.

**Table 2:** Properties of the Berea Sandstone

Porosity *= ϕ* (–)	Permeability *k*_0_ (mD)	Pore Throat Size^a^ Λ (in µm) (1)
0.206^b^	498^b^	7.9
0.205^b^	496^b^	7.9
0.207^b^	477^b^	7.7
0.180^c^	228^c^	6.0
0.230^d^	450^d^	6.8

a From 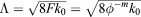 with *m* = 1.74.

b From *Aggour et al*. [1994].

c From *Lesmes and Frye* [2001].

d From *Zhu and Toksöz* [2012].

**Table 3:** Properties of the Sandstones From the *Vinegar and Waxman* [1984] Database Analyzed With the Linear Conductivity Model (Shaly Sands, NaCl)

Sample	Mineralogy^a^	*F* (–)^b^	*σ_S_*^c^ (10^−4^ S m^−1^)	*ϕ*(−)^d^	CEC^d^ (C kg^−1^)	log *Q_V_*^e^(C m^−3^)	*f*^f^ (–)
3477	K, C	15.1	30.6	0.201	237.74	6.40	0.85
3336A	K	23.0	23.6	0.205	393.65	6.61	0.93
3478	K, C	18.9	21.9	0.181	417.70	6.70	0.94
101	K, I	16.4	43.0	0.210	531.40	6.72	0.91
102	K, I	16.2	13.1	0.205	599.84	6.79	0.97
CZ10	C	17.8	72.6	0.233	772.91	6.83	0.89
3833A	C	13.9	60.7	0.224	1154.1	7.03	0.94
3126B	I, S	11.8	96.2	0.249	1446.1	7.06	0.92
3847A	I, C	47.7	29.6	0.126	754.56	7.14	0.96
3283A	K, C, I	19.4	44.0	0.186	1245.8	7.16	0.96
3885B	I, C	28.9	50.4	0.204	1676.7	7.24	0.97
3972E	S, M, I	21.7	44.8	0.187	1546.7	7.25	0.97
3258A	I	49.9	86.0	0.128	2022.1	7.56	0.95
3891A	C, I	39.3	222.4	0.192	3325.2	7.57	0.93
3308A	S, C	12.9	381.	0.270	5498.4	7.59	0.91
3323F	S, C	14.4	1065	0.288	10145	7.82	0.89
3324A	S, C	29.9	932	0.220	6622.6	7.79	0.84
3323E	S, C	15.1	1124	0.281	9801.6	7.82	0.87
3324B	S, C	29.6	907	0.204	7843.3	7.91	0.87
3306F	S, C	47.8	853	0.171	7122.6	7.96	0.87

a From *Vinegar and Waxman* [1984, Table 1], K: kaolinite, C: chlorite, I: illite, and S: smectite.

b From the fit of the in-phase conductivity data using the linear conductivity model.

c From the fit of the in-phase conductivity data using the linear conductivity model.

d From *Vinegar and Waxman* [1984].

e From *Q_V_* and porosity assuming a mass density for the grains of 2650 kg m^−3^.

f Using *f* = 1 − 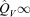/*Q_V_*.

## 5. High-Frequency Effective Permittivity

For frequencies much above the critical frequency 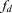 given by equation (82) and much below the relaxation frequency of water and bound water, the effective relative permittivity is given by


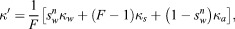
(104)

where 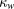 = 80 and 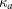 = 1 denote the relative permittivity of water and air, respectively. I now test the predictions of equation (104) with respect to saturation, porosity, and CEC.

### 5.1. Influence of Saturation

**Figure 11:**
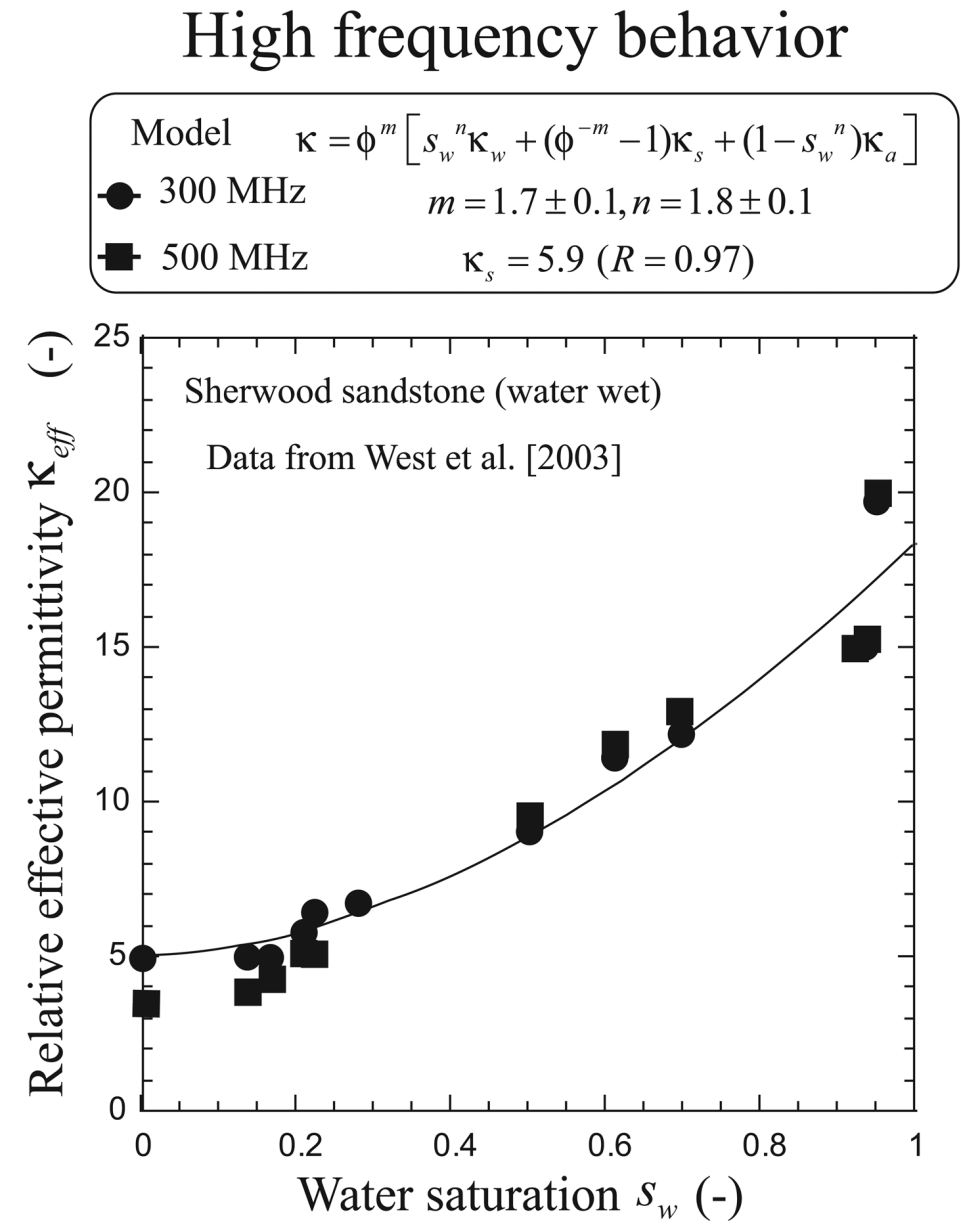
Saturation dependence of the effective permittivity. Relative effective permittivity at very high frequencies (300 and 500 MHz) for the Sherwood sandstone (porosity 0.36, data from *West et al*. [2003]. The plain line corresponds to the prediction of the model.

I use the data from *West et al*. [2003] at 300 and 500 MHz, therefore, below the dielectric relaxation frequency of water. A fit of the data (shown in Figure 11) yields *m* = 1.7 ± 0.1 and *n* = 1.8±0.1 (for the same sandstone, *Binley et al*. [2002] obtained from low-frequency electrical conductivity measurements *m* = 1.7 and *n* = 2.0, respectively), and a relative permittivity for the solid phase of 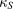 = 5.9 (in agreement with the range provided by *Robinson and Friedman* [2003] for similar sandstones).

### 5.2. Influence of Porosity

**Figure 12:**
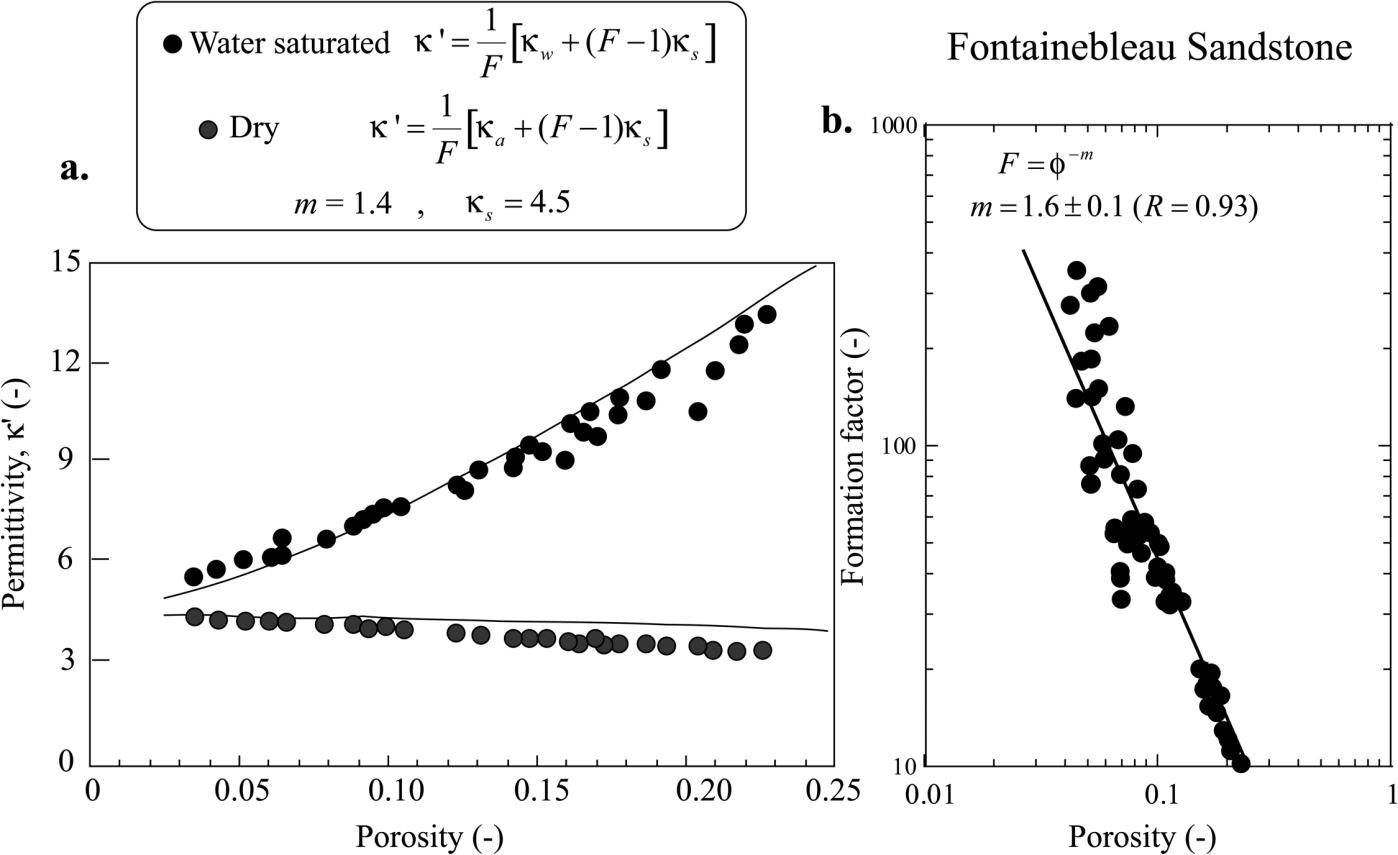
High-frequency relative permittivity of the Fontainebleau sandstone. (a) Permittivity measurements between 50 MHz and 1 GHz (data from *Coutanceau-Monteil* [1989]). The lines represent the prediction of the model. (b) Formation factor/porosity relationship (Zamora et al., unpublished results). The plain lines correspond to the prediction of the model.

**Figure 13:**
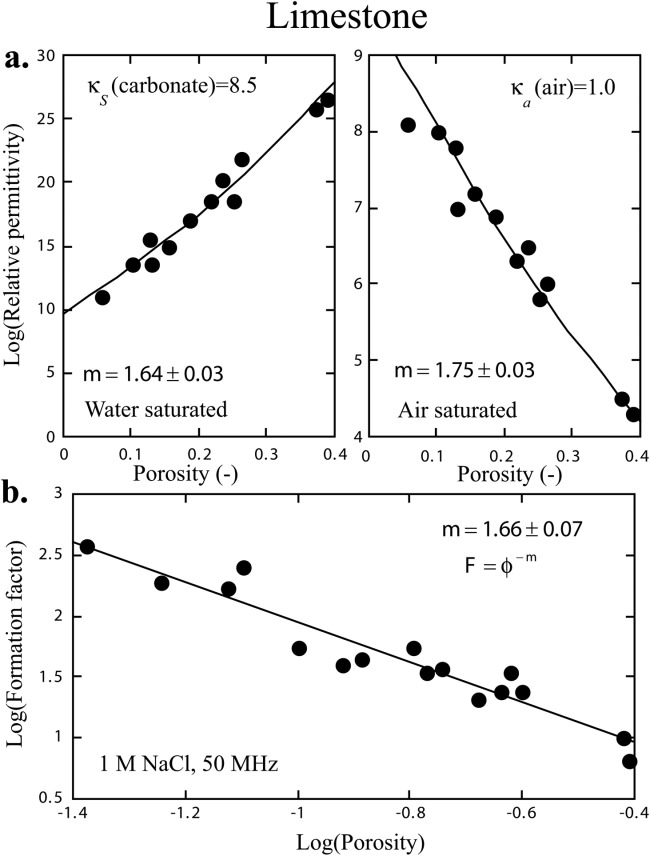
Comparison between the model and the experimental data from *Coutanceau-Monteil* [1989] for limestones. (a) The samples are saturated by fresh water and air. (b) Calibration of Archie's law from the measurement of the formation factor and porosity for samples of the same formation than used for the relative permittivity (50 MHz). The plain lines correspond to the prediction of the model.

In Figure 12, I test the effect of porosity upon the high-frequency relative permittivity. I use the data from *Coutanceau-Monteil* [1989] with the Fontainebleau sandstone (99.98% silica). The measurements were performed at high frequencies between 50 MHz and 1 GHz. The lines in Figure 12 represent the prediction of the model. The application of the model to the data yields a cementation exponent of 1.5 and a relative permittivity for the solid phase of 4.5. This relative permittivity is very close to the relative permittivity for SiO_2_ (3.9 according to *Gray et al*. [2009] and 4.5 according to *Olhoeft* [1981]). The cementation exponent is also consistent with electrical conductivity measurements (Figure 12b). In Figure 13, the prediction of equation (104) for the relative permittivity/porosity relationship is tested for carbonate rocks. The model is consistent with the data and the value of the cementation exponent determined from electrical conductivity measurements.

### 5.3. Influence of CEC

**Figure 14:**
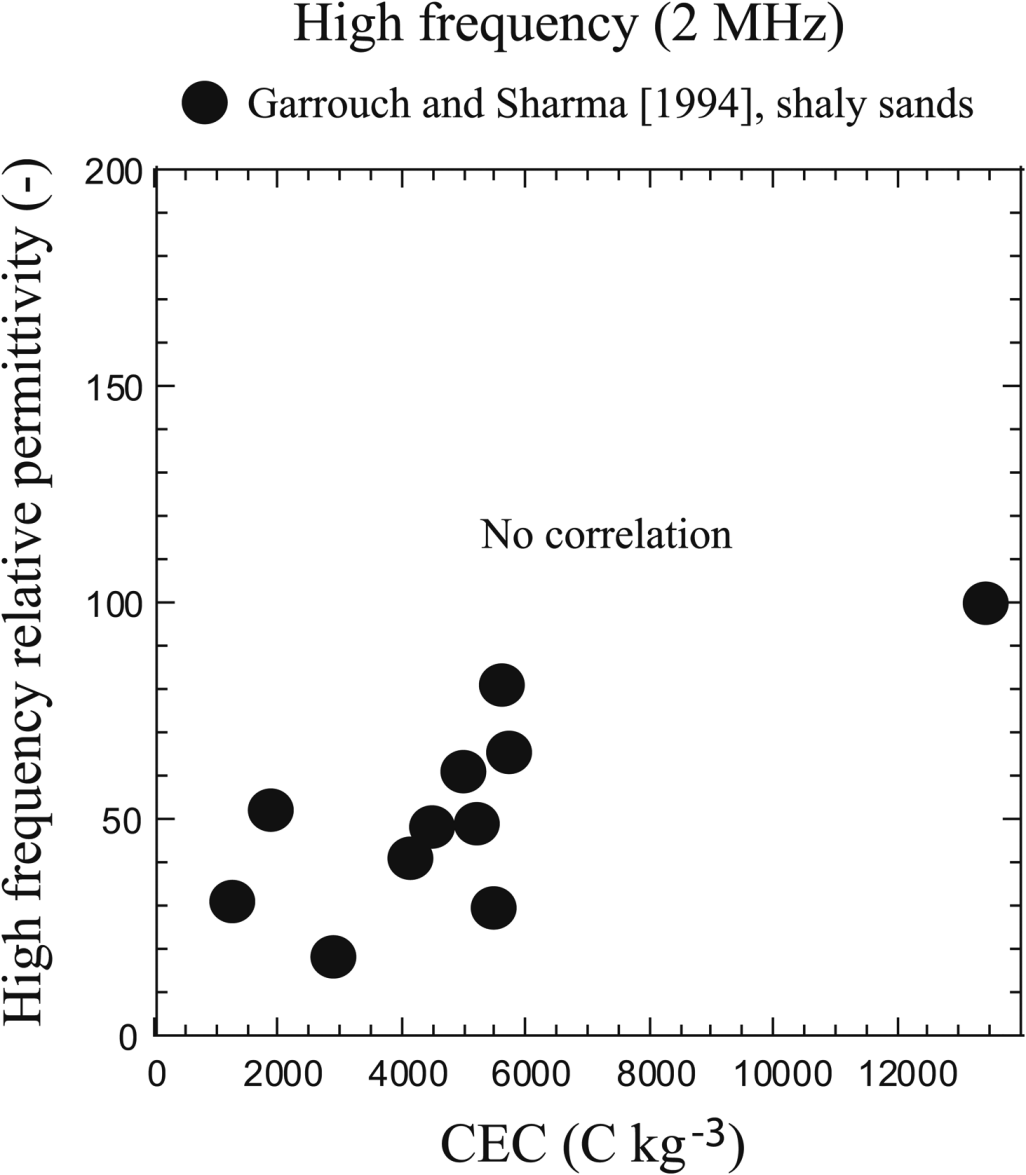
At high frequencies (2 MHz), the effective permittivity appears independent (or weakly dependent) on the CEC, a result that is also in agreement with the model developed in the present paper [*Garrouch and Sharma*, 1994].

According to equation (104), the high-frequency relative permittivity should be independent on the cation exchange capacity. Figure 14 displays various experimental data and seems to show that this is the case.

## 6. Relaxation Time, Pore Size, and Permeability

**Figure 15:**
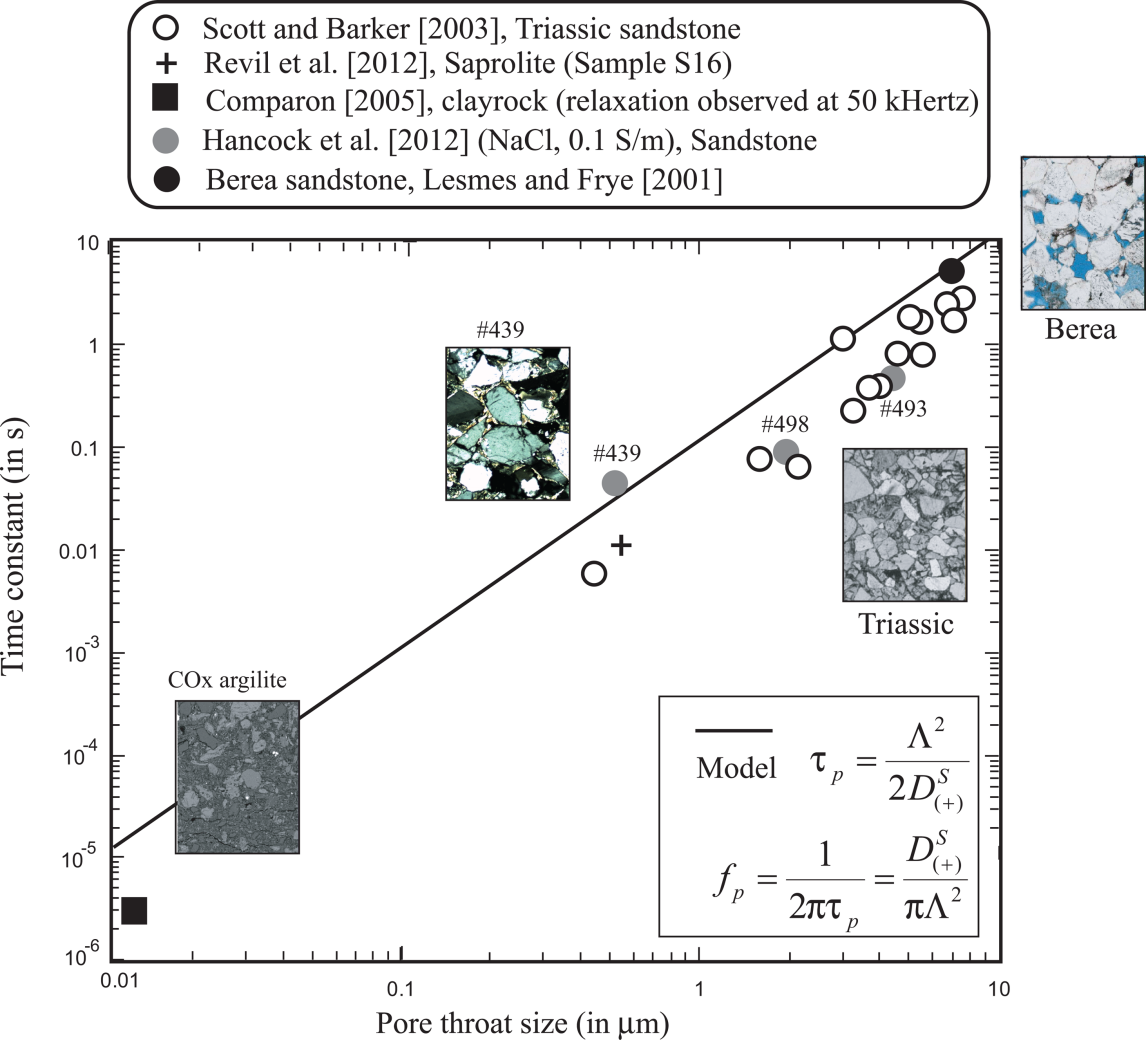
Relationship between the low-frequency time constant and the mean size of the pore throat for clayey materials. The size of the thin section images is 1 mm in *x*. COx stands for Callovo-Oxfordian. The plain line corresponds to the prediction of the model.

**Figure 16:**
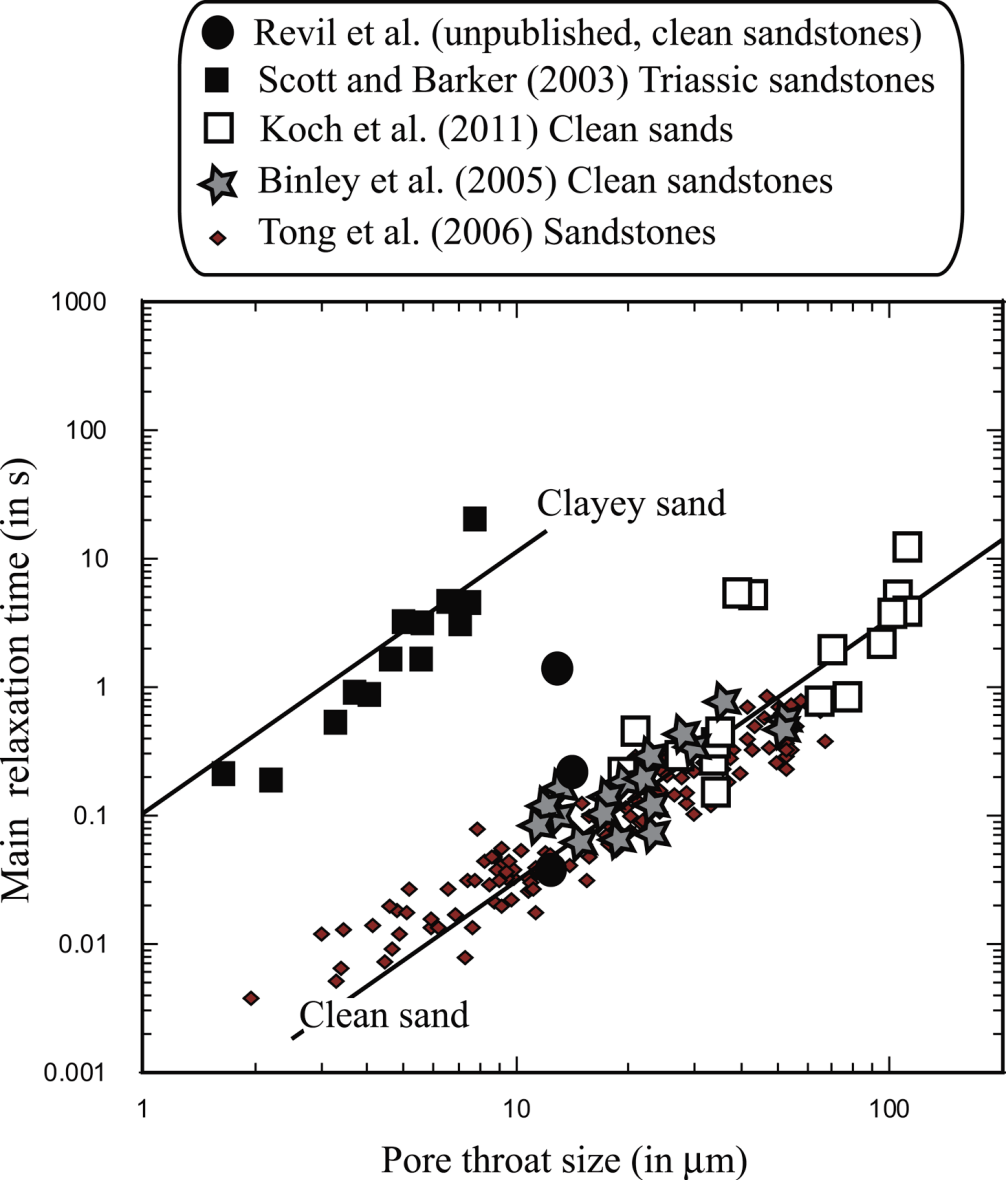
Main relaxation time *τ*_0_ versus the pore size Λ. The pore size data from this study are median values obtained from mercury injection data or permeability data. For the data from *Koch et al*. [2011], the pore size is determined from the median grain size and the formation factor using the relationship developed by *Revil and Florsch* [2010]. The mean pore size is either determined from mercury intrusion porosimetry [*Binley et al*., 2005] or from permeability for the data of *Tong et al*. [2006]. The plain lines correspond to the prediction of the model.

In Figures 15 and 16, I test the relationship between the relaxation frequency and the pore throat size given by equation (78) for clayey sands and clean sandstones, respectively. For the Triassic sandstones reported by *Scott and Barker* [2003], I divided the mercury pore diameter by 2 to get a mercury pore access and then I divided the result by 5.3 to get a value of Λ. Indeed, the capillary entry pressure is related to the mercury pore radius *r_c_* by 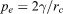, where *γ* represents the surface tension and *r_c_* represents the smallest pore of the set of largest pore percolating through the porous material. *Katz and Thompson* [1987] developed a relationship between the permeability and the percolation length scale *r_c_* using percolation principles: 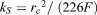. A comparison between this equation and equation (A11) of Appendix B yields *r_c_* ≍ 5.3 Λ.

**Figure 17:**
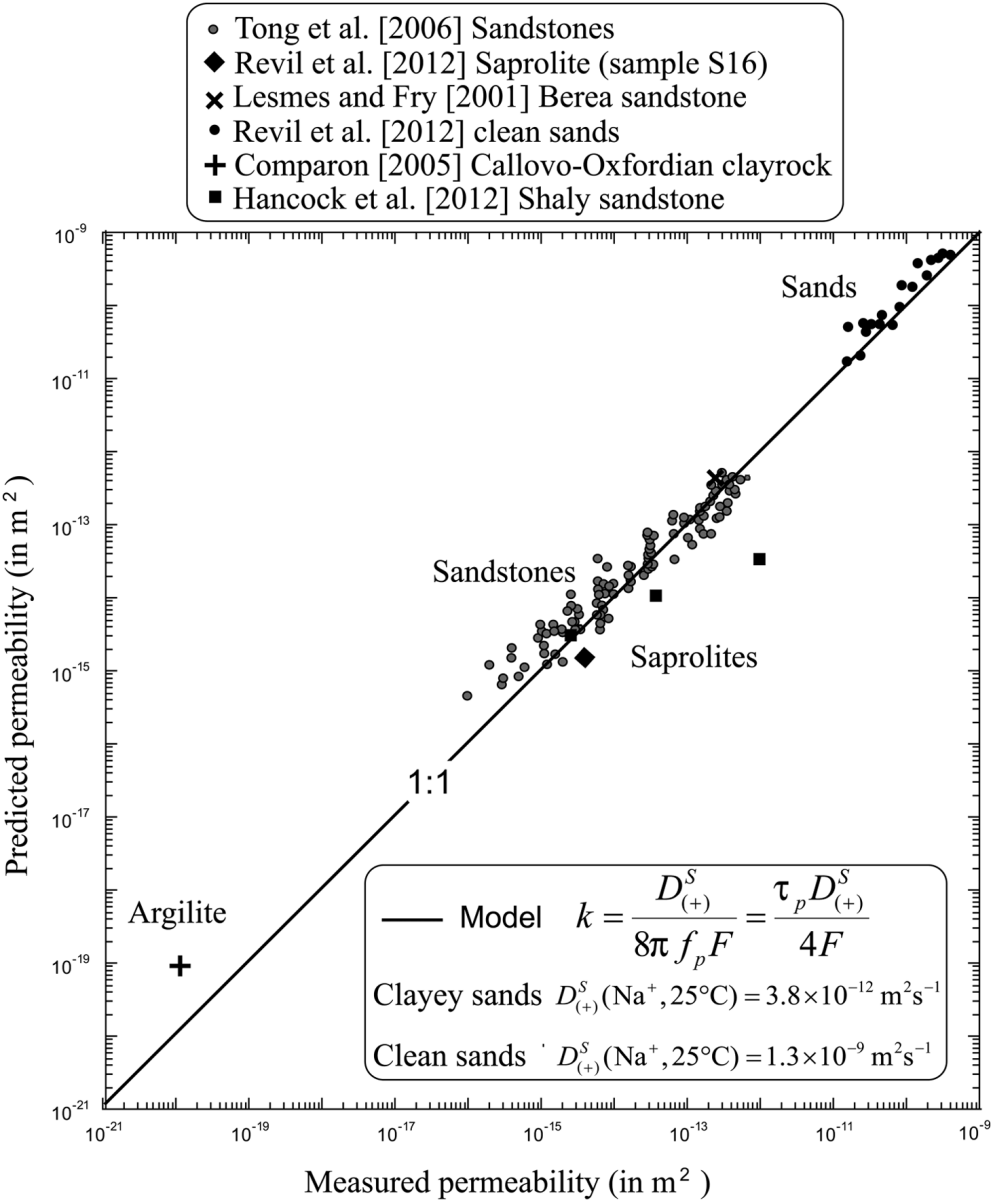
Predicted versus measured permeability for various clean sands and clayey sands and sandstones using the low-frequency relaxation time *τ_p_* or the Cole-Cole relaxation time.

It seems that the proposed relationship works well. There is however a need for further investigations to confirm this finding, especially for clayey materials. In Figure 17, I use equation (79) to predict the permeability from the low-frequency relation time *τ_p_* and the formation factor *F*. The results show that the permeability can be pretty well predicted inside an order of magnitude from the complex conductivity. The data covers 11 orders of magnitude. Note that the diffusivity of the counterions for clean sands is equal to the diffusivity of the counterions in the bulk pore water (see discussion in *Revil et al*. [2012a]) while it is 2 orders of magnitude smaller for clays [see *Revil*, 2012].

## 7. Relationship Between *m*, *n*, and *p*

To reduce the number of input parameters to bridge reactive transport modeling and geophysical predictions, I look now for some empirical relationships between the three exponents used in the model. The cementation exponent *m* can be determined from the cation exchange capacity according to


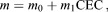
(105)

with *m*_0_ = 1.80 and *m*_1_ = 4.3 × 10^−5^ kg C^−1^ and the CEC is expressed in C kg^−1^ [*Revil et al*., 1998]. We test the two relationships to predict the saturation exponent from the cementation exponent. The first one is purely empirical and has no theoretical foundations but is broadly used in the literature in absence of independent estimates of *n*:


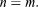
(106)

**Figure 18:**
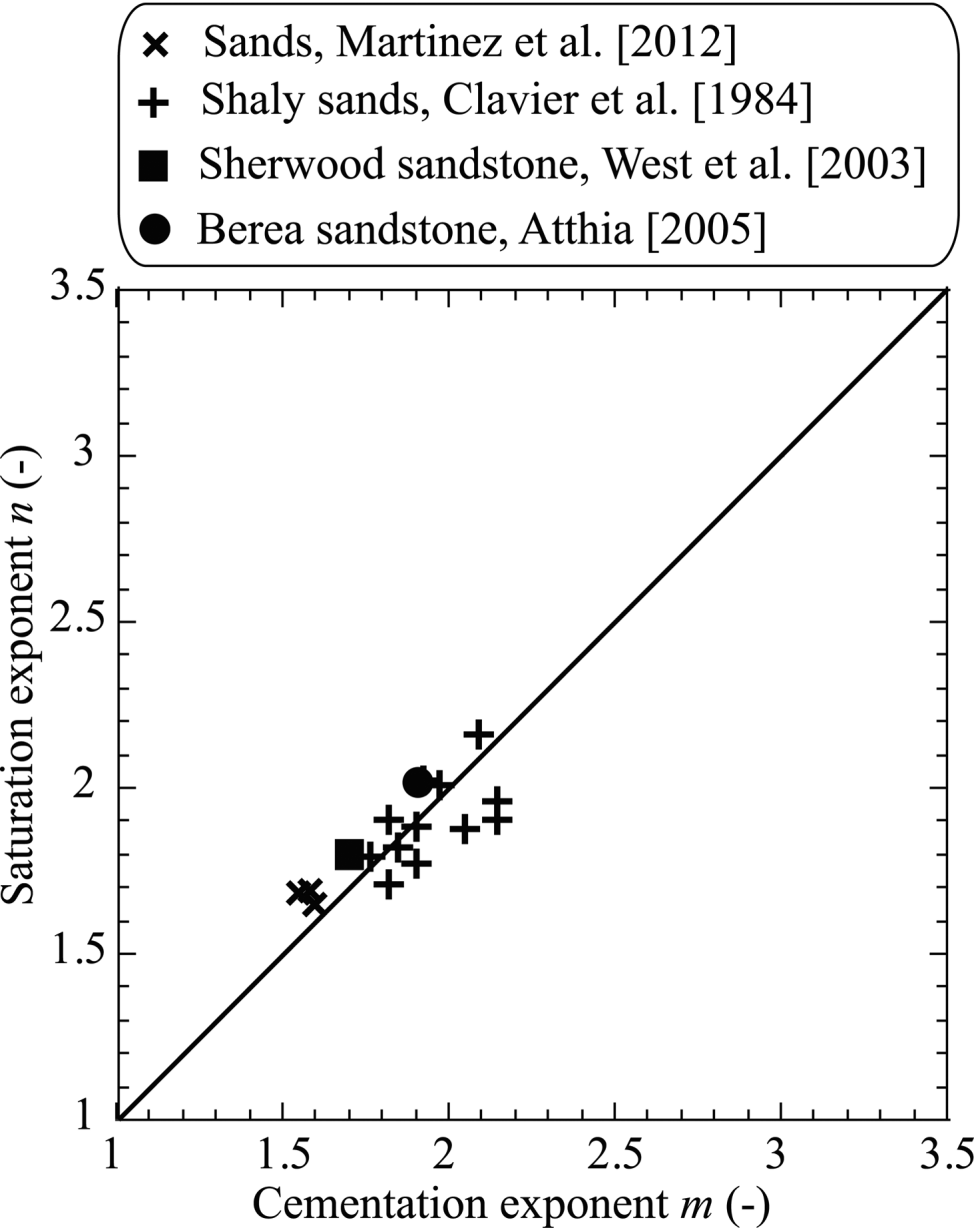
Saturation versus cementation exponent (*n* versus *m*). Data from *Clavier et al*. [1984], *West et al*. [2003], *Atthia* [2005], and *Martinez et al*. [2012]. All these data are corrected for the effect of surface conductivity when such a correction is required.

This relationship is evaluated in Figure 18. Note that a number of published papers, in which the two Archie exponents *m* and *n* are reported, cannot be used to assess equation (106). Indeed, in these papers, surface conductivity is not taken into account and the reported values of *m* and *n* may be, therefore, grossly wrong. Figure 18 seems to indicate that the approximation 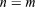 is good enough to be used in reactive transport modeling (at least in a stochastic sense with an associated probability density).

**Figure 19:**
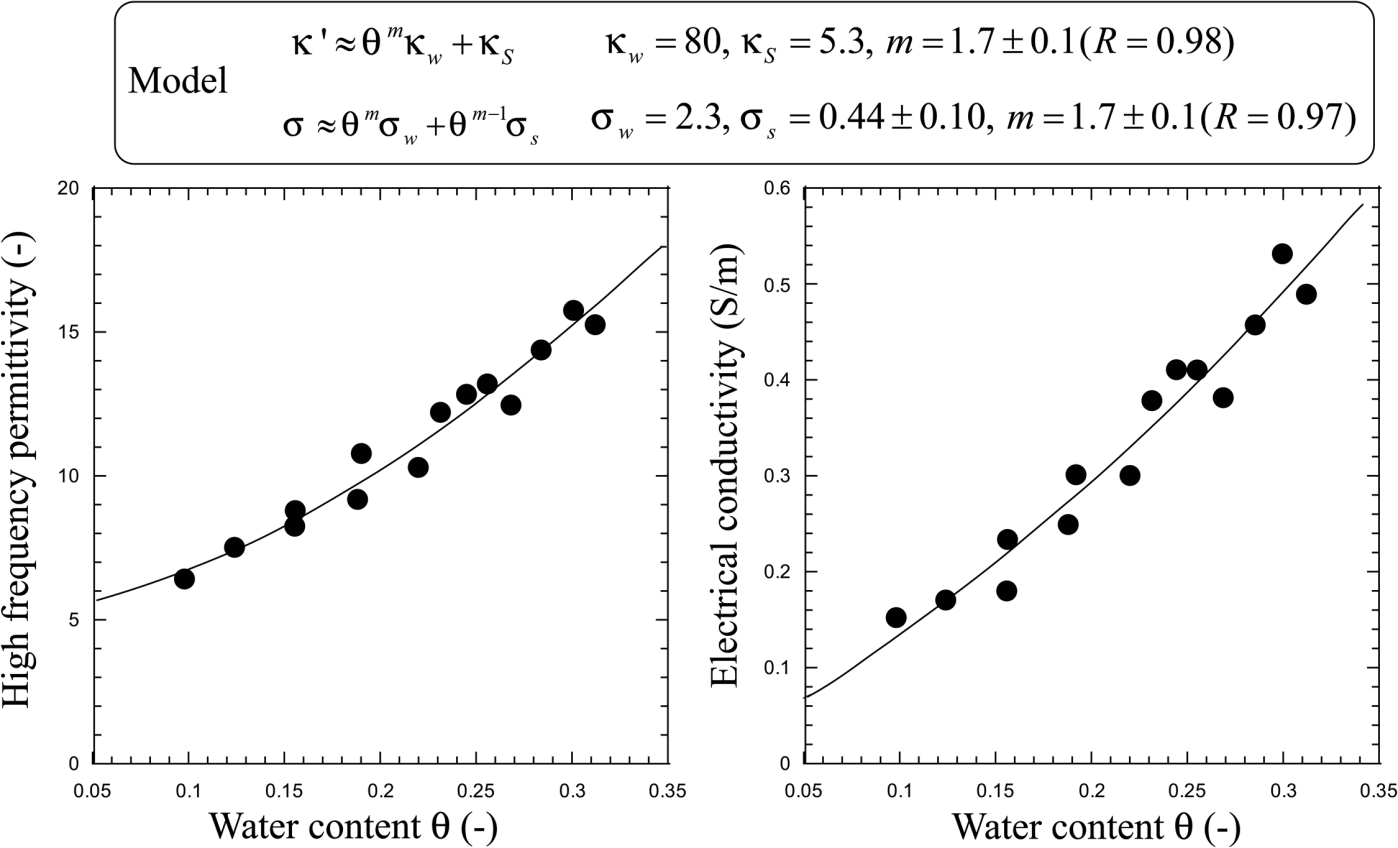
Comparison between the simplified permittivity and conductivity models (expressed as a function of the water content, see equations (107) and (108)) and experimental data from *Comparon* [2005] at 1.3 GHz for mixtures of MX80(bentonite)/kaolinite. Porosity 0.40.

The approximation 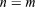 also provides a simple equation to predict the water content from the permittivity. Taking indeed *m* = *n* and neglecting the contribution from the air, the permittivity of the air yields


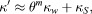
(107)


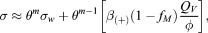
(108)

which is checked in Figure 19 for a bentonite-kaolinite mixture.

We can also look for a way to predict the quadrature exponent from the saturation exponent. Following *Vinegar and Waxman* [1984], *Revil* [2012], and section 2 above, I can test the following relationship,


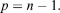
(109)

**Figure 20:**
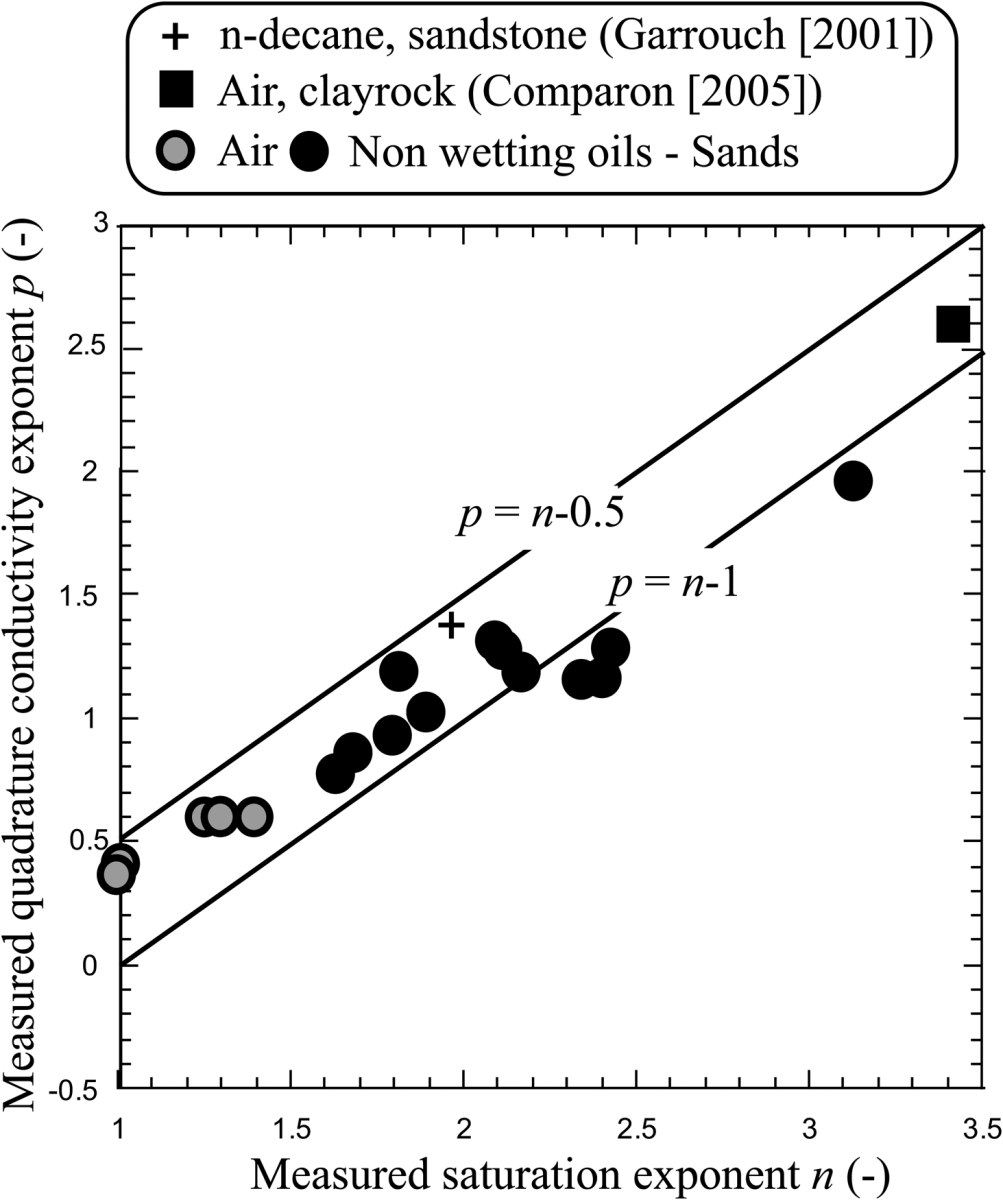
Saturation exponent *p* of the quadrature conductivity/effective relative permittivity versus the second Archie exponent *n* for the in-phase conductivity. The sand data are from *Schmutz et al*. [2012], *Vinegar and Waxman* [1984], *Ulrich and Slater* [2004], and *Abdel Aal et al*. [2006]. Other data are from *Garrouch* [2001] and *Comparon* [2005].

In Figure 20, I plot various data from the literature for which *p* and *n* were independently determined. It seems that the quadrature conductivity exponent *p* is comprised between (*n* − 1) and (*n* − 0.5).

If we account for equations (104) to (106), the reduction in the number of the parameters is such that the porosity, a pore throat size, and the CEC of the material are the only three fundamental textural parameters required to predict the complete electrical response of the clayey materials in the frequency range 1 mHz–1 GHz. Of course, the environmental parameters (including the salinity and the pH of the pore water and the temperature) also control some of the parameters used in my model. However, these dependencies are well established.

## 8. Concluding Statements

A simple model has been developed to predict the apparent conductivity and permittivity (or alternatively the apparent quadrature conductivity) as a function of the frequency, clay content, and clay mineralogy. This model has been tested on a broad number of experimental data and seems to explain consistently the available data pretty well. It also offers the possibility to predict permeability from the critical frequency observed at low frequencies in the quadrature conductivity. My goal with this model is to start to have a unified petrophysical framework that can be used to develop time-lapse joint inversion algorithms for DC resistivity, induced polarization, induction-based EM, seismoelectricity, and GPR for shallow subsurface applications, especially regarding the vadose zone and agriculture.

## Appendix A: Saturation Dependence with the DEM Approach

The differential effective medium (DEM) approach treats the fluid and solid phases in an asymmetric way: one of the two phases has to be the host for the other (for instance, grains immersed in a background electrolyte). This approach therefore offers a view that complements the volume-averaging approach discussed in the main text. The solution of the differential effective medium scheme yields the following expression for the electrical conductivity [*Sen et al*., 1981]:

(A1)

(where we have replaced *F* of the saturated case by ). Equation (A1) has, at low and high frequencies, the following closed-form solutions [*Revil et al*., 1998]

(A2)

(A3)

The Dukhin number is defined by [*Revil et al*., 1998; *Revil*, 2012]

(A4)

Therefore, using equations (65) and (66) of the main text (with  = 1), we have

(A5)

(A6)

An equation similar to equation (A1) can be found for the permittivity,

(A7)

where  denotes the permittivity of the pore fluid. An approximation of this equation is

(A8)

where . Now, for unsaturated materials we can used the following relationship,

(A9)

Therefore, the saturation dependence of  is

(A10)

## Appendix B: Relaxation Time Versus Saturation

We define in this section the two textural parameters *F* and Λ. The following canonical boundary value problem for the normalized potential Γ for a cylindrical REV of porous material of length *L* can be written as (see *Pride* [1994])

(B1)

(B2)

(B3)

where *z* denotes the distance along the axis of the cylindrical core. In these equations,  denotes the unit vector normal to the pore water/mineral interface *S* and *V_p_* corresponds to the pore volume. The boundary conditions defining the normalized potential Γ are representative for the injection of an electrical current into a rock sample in the absence of surface conduction along the pore/water interface [see *Johnson et al*., 1986; *Avellaneda and Torquato*, 1991; *Pride*, 1994]. The dynamic pore radius Λ and the formation factor *F* are defined as [*Johnson et al*., 1986]

(B4)

(B5)

where *V* is the total volume of the considered REV. The length scale Λ therefore corresponds to a weighted version of the hydraulic pore radius *V_p_*/*S* weighted by the norm of the electrical field (normalized by the electrical field imposed from the boundary of the material) and therefore Λ can be seen as the (dynamic) characteristic pore size of the material.

Using a volume-averaging approach, *Johnson et al*. [1986] and *Pride* [1994] developed the following general equation for the electrical conductivity of a water-saturated rock:

(B6)

where Λ is a characteristic pore length scale defined above and  denotes the specific surface conductivity associated with the diffuse part of the electrical double layer (in S). Similarly the high-frequency conductivity and the normalized chargeability should be obtained by

(B7)

(B8)

where  denotes the specific surface conductivity associated with the Stern layer (in S). To be compatible with equations (72) to (74), the following scaling laws for the dependence of the formation factor and length scale Λ with the relative water saturation should hold,

(B9)

(B10)

The quasi-static permeability  (for *ω* = 0) is related to the formation factor *F* and the dynamic pore radius  as [*Johnson et al*., 1986],

(B11)

Therefore, the permeability should scale with the water saturation according to the following power law,

(B12)

According to this scaling, the permeability can be computed as the product of a permeability at saturation *k_S_* and a relative permeability that depends only on the relative water saturation  with . This type of power-law behavior is also the one predicted by the *Brooks and Corey* [1964] model.

The relaxation time for the low-frequency polarization is given by [*Revil et al*., 2012a]

(B13)

Consequently, using equation (B10), this yields the following dependence of the relaxation time with saturation,

(B14)

This equation provides a way to compute the low-frequency relaxation time from the pore size of the throat and from the saturation.
